# Supplementary material for: A polymer tethering strategy to achieve high metal loading on catalysts for Fenton reactions
Source: Nat Commun. 2023 Nov 29;14:7841. doi: 10.1038/s41467-023-43678-1 (PMC10687042; doi:10.1038/s41467-023-43678-1)
Supplement: Supplementary file 1 — Supplementary Information [file 41467_2023_43678_MOESM1_ESM.pdf]

Supplementary Information

A polymer tethering strategy to achieve high metal loading on catalysts for Fenton reactions

Lixin Wang[1], Longjun Rao[1], Maoxi Ran[2], Qikai Shentu[1], Zenglong Wu[1], Wenkai Song[1], Ziwei Zhang[1], Hao Li[1], Yuyuan Yao[1,3], Weiyang Lv[1,3] and Mingyang Xing[1,2]**

[1]National Engineering Lab of Textile Fiber Materials & Processing Technology (Zhejiang), Zhejiang Sci-Tech University, Hangzhou 310018, China

Corresponding authors: wylv@zstu.edu.cn; mingyangxing@ecust.edu.cn

[2]National Engineering Research Center of Industrial Wastewater Detoxication and Resource Recovery, School of Resources and Environmental Engineering, East China University of Science and Technology, 130 Meilong Road, Shanghai 200237, China

Corresponding author: mingyangxing@ecust.edu.cn

[3]Zhejiang Provincial Innovation Center of Advanced Textile Technology, Shaoxing 312000, China

Corresponding author: wylv@zstu.edu.cn

Experimental Section

Chemicals

Dopamine hydrochloride (DA, 98%), iron chloride hexahydrate ($FeCl_3•6H_2O$), cobalt chloride hexahydrate ($CoCl_2•6H_2O$), nickel chloride hexahydrate ($NiCl_2•6H_2O$), copric chloride dihydrate ($CuCl_2•2H_2O$), silver chloride (AgCl), zero valent iron (ZVI), and 5,5-dimethyl-1-pyrroline N-oxide (DMPO, 97%) were obtained from Shanghai Macklin Biochemical Co., Ltd.. Sulfamethoxazole (SMX), carbamazepine (CBZ), diclofenac (DCF), ciprofloxacin (CIP), enrofloxacin (ENR), atrazine (ATZ), bisphenol A (BPA), methyl alcohol (MA, 99%), tert-butyl alcohol (TBA, 98%), p-benzoquinone (p-BQ), phthalhydrazide (phth, 99%), phosphoric acid (H_3PO_4, 99%), dimethyl sulfoxide (DMSO, 99%), catalase (200000 unit g^{-1}), humic acid (HA) and iron (III) acetylacetonate ($Fe(acac)_3$) were purchased from Aladdin Reagent Co., Ltd. (Shanghai, China). Sulfuric acid (H_2SO_4, 98%) and hydrogen peroxide solution (H_2O_2, 30 wt%) were supplied by Zhejiang Sanying Chemical Reagent Co., Ltd.. All other agents included in the study were acquired from Hangzhou Mike Chemical Instrument Co., Ltd. (Hangzhou, China). Unless otherwise stated, all agents were analytical grade and were used as received without further purification. Ultrapure water was employed to prepare all aqueous solutions in this study, the characteristics of the water matrix were listed as follows:

**Item**	**Standard requirement**
Chromaticity (°)	＜5
Turbidity (NTU)	＜1
Smell and taste	no
pH	6.0~7.0
Conductivity (μS cm^{-1})	＜5

Electron paramagnetic resonance (EPR) measurement

The EPR spectra were recorded via a Bruker A300 spectrometer with DMPO as spin-trapping agent to trap the reactive oxygen species. For detecting $^{\bullet}OH$, the reaction was initiated by adding a certain dosage of catalyst sample and H_2O_2 into a 100 mL glass flask containing 20 mL deionized water. At predetermined time intervals, 1 mL reaction solution was withdrawn and immediately quenched with 20 μL DMPO (97%). The parameters of the EPR spectrometer were as follow: center field, 3520 G; sweep width, 100 G; microwave frequency, 9.77 GHz; modulation frequency, 100 KHz; power, 12.72 mW.

Fluorescence monitoring

The fluorescence images of hydroxyl radicals were detected on a laser scanning fluorescence microscope with phthalhydrazide as the chemiluminescence probe. Typically, 2.5 mg catalyst was dispersed in the 50 mL phthalhydrazide solution (0.1 M), and the reaction was initiated by adding 5×10^{-6} L H_2O_2 (30 wt%). At a given time, a certain volume reaction solution was withdrawn and immediately monitored on the laser scanning fluorescence microscope.

Operation of the phosphoric acid poisoning experiment

The pyridinic-N sites could be protonated after adding phosphoric acid (H_3PO_4), in which the pyridinic-N-H (a pyridinium) structure would be formed. The H_3PO_4 treatment experiments were carried out in a 100 mL conical flask containing 20 mL organic pollutant aqueous solution. An appropriate amount of phosphoric acid solution was added into reaction system before the addition of catalyst and oxidant. The pH of system was not adjusted after adding H_3PO_4. Other

reaction conditions and steps remained consistent with pollutant degradation experiments.

Operation of quantification analysis of surface-bound $^{\bullet}OH$

Firstly, we added F^{-} in the solution to desorb $^{\bullet}OH_{surface}$ by forming strong $^{\bullet}OH_{surface}\cdots F^{-}$ hydrogen-bond. Then, the cumulative concentration of $^{\bullet}OH$ was quantified by indirect detection of hydroxybenzoic acid (HBA) since $^{\bullet}OH$ could be captured by excess benzoic acid (BA), leading to the generation of HBA. The generated HBA during the reaction was detected by high performance liquid chromatograph (HPLC), and other steps were consistent with the catalytic experiment procedure. The relative ratio of different types of $^{\bullet}OH$ was calculated by comparing the HPLC peak areas of HBA before and after the addition of F^{-}.

Contaminant concentration assessment

The varied concentrations of organic pollutants were analyzed by high performance liquid chromatograph (HPLC) equipped with Waters 2998 Photodiode Array, which offered advanced optical detection and trace quantitation of sample in conjunction with spectral analysis capability. The operation details, detection wavelengths and mobile phase of different organic pollutants were listed in Supplementary Table 8 and Table 9. The variations of organic dyes were analyzed with UV-Vis spectrometer (Unico V-1800) at the maximum absorption peak, and the maximum absorption wavelengths of corresponding dyes were listed in Supplementary Table 10.

Water quality monitoring

The leached Fe ions during reaction solution were detected on an inductively coupled plasma optical emission spectrometer (ICP-OES, PerkinElmer 8300). Total organic carbon (TOC) contents were analyzed on a TOC detector (Shimadzu TOC-L). Chemical oxygen demand (COD) contents were measured on a COD detector (Lianhua 5B-3F).

Calculation of H_2O_2 effective utilization efficiency

In stoichiometry, complete mineralization of one mole SMX will consume 33 mole H_2O_2 (Equation 1).

$$C_{10}H_{11}N_3O_3S+33H_2O_2\rightarrow10CO_2+36H_2O+3HNO_3+H_2SO_4 \quad (1)$$

Therefore, the H_2O_2 effective utilization efficiency (η) was defined as Equation 2:

$$\eta = [\Delta H_2O_2]_S \,/\, [\Delta H_2O_2]_C \quad (2)$$

where $[\Delta H_2O_2]_S$ referred to the stoichiometric amount of H_2O_2 for SMX complete mineralization, $[\Delta H_2O_2]_C$ was the total real consumed amount of H_2O_2 during the reaction. The real consumed amount of H_2O_2 was monitored by titanium potassium oxalate colorimetry.

Kinetics analysis

The reaction rate was calculated by a pseudo-first-order kinetics model (Equation 3), and the contaminant removal was expressed as Equation 4:

$$\ln(C_0/C_t) = K_{obs} \times t \quad (3)$$

$$\text{contaminant removal} = (C_0\text{-}C_t)/C_0 \times 100\% \quad (4)$$

where C_0 was the initial concentration of contaminants, C_t referred to the concentration at a

certain time t during the catalytic reaction process and the apparent reaction constant K_{obs} was evaluated from the slopes of plot of ln (C_0/C_t) versus time.

Characterization

Scanning electronic microscopy (SEM) images were detected on a SEM microscope (Zeiss Gemini 300, 5 - 20 kV). Transmission electronic microscopy images were obtained on a TEM microscopy (JEOL JEM 2100, 200 kV). Atomic force microscopy (AFM) images were performed by an AFM microscope XE-100E in the air condition. Powder X-ray diffraction (XRD) patterns were recorded on an X-ray diffractometer (BrukerAXS D8 advance) equipped with Cu Kα radiation (λ = 1.5406 Å) at a voltage of 40 kV and a current of 40 mA with a scanning speed of 5° min^{-1}. Room-temperature ^{57}Fe Mössbauer spectra of the samples were measured in a Mössbauer spectrometer working in constant acceleration mode and equipped with a ^{57}Co (pd) γ-rays source. The recorded ^{57}Fe Mössbauer spectra were analyzed with the least-square methods. X-ray photoelectron spectroscopy (XPS) spectra were collected on an X-ray photoelectron spectrometer (Thermo Kalpha+) equipped with an excitation source of monochromatic aluminum, and the Ar ion was used to etch the catalyst surface. The C 1*s* value was set at 284.8 eV for corrections, and the XPS spectra were analyzed by XPSPEAK41 software with Shirley-type background. The Brunner-Emmet-Teller (BET) specific surface area of samples were evaluated by Micromeritics TriStar II at 77 K in the N_2 conditions. Raman spectra were collected on LabRAM Horiba Evolution with a 532 nm excitation laser. Inductively coupled plasma optical emission spectrometer (ICP-OES) was used to measure the content of metal in the sample by PerkinElmer 8300. Molecular weights and molecular weight

distributions of Fe-PDA complex were determined by gel permeation chromatography (GPC) using Agilent PL-GPC50. Thermogravimetry analysis (TGA) was carried on Jupiter STA 449 F1 under N_2 atmosphere.

Density Functional Theory computation details

All the DFT computations were conducted adopting the Vienna Ab initio Simulation Package (VASP). The interactions of ion-electron were depicted using the projector augmented wave approach and the general gradient approximation in the Perdew-Burke-Ernzerhof (PBE) Form was applied. The convergence criteria were set to 0.03 eV $Å^{-1}$ and 10^{-5} eV for the residual force and energy during structure relaxation. The plane-wave cutoff energy was set as 520 eV, and 3×3×1 k-points were served as sample for the Brillouin region. Other than these, we had set a vacuum region of 15 Å along the z-direction to avoid interactions between adjacent images. For adsorption studies, a 2×2×1 supercell was built for Fe (110) and graphene (002) plane. The Gibbs free energy change (ΔG) for each elementary step was obtained by $\Delta G = \Delta E + \Delta E_{ZPE} + \Delta\int Cp\mathrm{d}T - T\Delta S$, where ΔE was electronic reaction energy, ΔE_{ZPE} and ΔS were the difference of zero–point energy correction and entropy, $\Delta\int Cp\mathrm{d}T$ was enthalpy correction, and T was 298.15 K. The climbing image nudged elastic band method (CI-NEB) was adopted to determine the minimum energy paths and to calculate energy barrier.

Results and discussion

Text 1. Analysis of low utilization efficiency of H_2O_2 in conventional Fenton system

In the conventional Fenton system (Fe^{II}/H_2O_2), an abundance of Fe^{II} ions could rapidly react with H_2O_2 to generate high concentration of $^{\bullet}OH$ within a short time (Equation 5). However, these $^{\bullet}OH$ would not only participate in the degradation of target pollutants, but also induce undesirable side reactions. These side reactions were the main reasons for the low utilization efficiency of H_2O_2: (i) Fe^{II} would react with $^{\bullet}OH$ to generate Fe^{III}, resulting in ineffective consumption of Fe^{II} and $^{\bullet}OH$ (Equation 6). (ii) Fe^{III} would react with H_2O_2 to generate $^{\bullet}O_2H$ (Equation 7). Then, the self-reaction between $^{\bullet}O_2H$ would generate O_2, resulting in ineffective consumption of H_2O_2 (Equation 8). (iii) Significant self-quenching reaction occurred when two $^{\bullet}OH$ met together (Equation 9), leading to a quick decrease in the concentration of $^{\bullet}OH$. In contrast, the Fe@N-C-800/H_2O_2 system had fewer side reactions due to the confinement of $^{\bullet}OH$ on carbon surface, thus avoiding the reaction of $^{\bullet}OH$ with Fe^{II} and itself. Moreover, the generation of $^{\bullet}O_2H$ was also alleviated in Fe@N-C-800/H_2O_2 system (Supplementary Fig. 29), which could also increase the utilization efficiency of H_2O_2.

$$Fe^{II} + H_2O_2 \rightarrow Fe^{III} + OH^{-} + {}^{\bullet}OH \quad (5)$$

$$Fe^{II} + {}^{\bullet}OH \rightarrow Fe^{III} + OH^{-} \quad (6)$$

$$Fe^{III} + H_2O_2 \rightarrow Fe^{II} + {}^{\bullet}O_2H + H^{+} \quad (7)$$

$${}^{\bullet}O_2H + {}^{\bullet}O_2H \rightarrow H_2O_2 + O_2 \quad (8)$$

$${}^{\bullet}OH + {}^{\bullet}OH \rightarrow H_2O_2 \quad (9)$$

Supplementary Figures

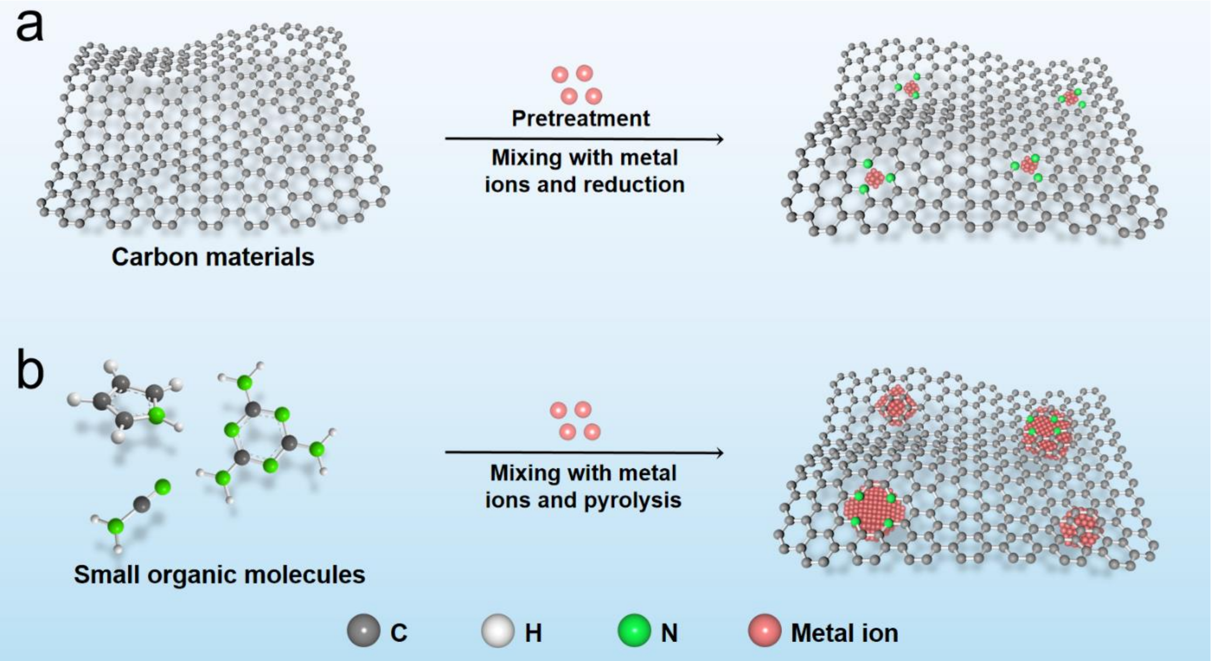

Supplementary Fig. 1 | Schematic illustration of different synthesis strategies to fabricate M@N-C. a The top-down strategy and **b** the bottom-up strategy.

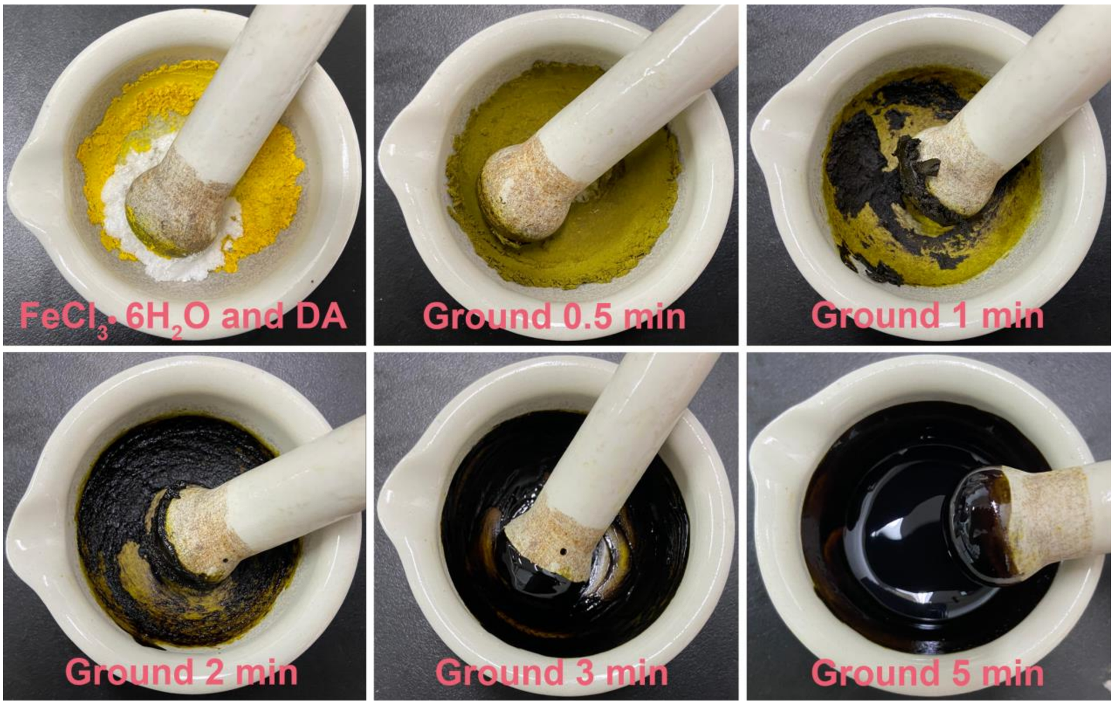

Supplementary Fig. 2 | Time course of the evolution of Fe-PDA complex during mixing and grinding.

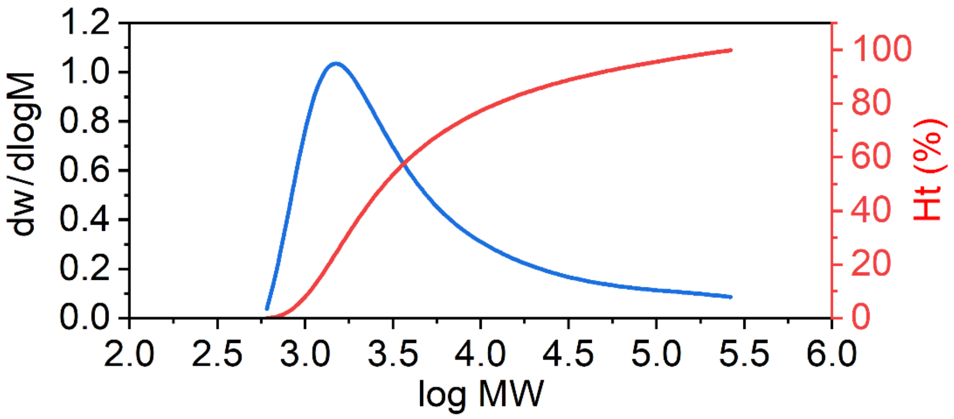

Supplementary Fig. 3 | GPC data of the Fe-PDA complex under the normal grind-assisted polymerization conditions.

MW Averages

Peak No	Mp	Mn	Mw	Mz	Mz+1	Mv	PD
1	1502	2321	**14787**	98983	160649	9802	6.37096

*Dimethyl sulfoxide (DMSO) was used as the solvent. The Mp, Mn, Mw, Mz, Mz+1 and Mv in the table are the common indexes for molecular weight evaluation, which represent peak molecular weight, number-average molecular weight, weight-average molecular weight, z-average molecular weight, z+1 average molecular weight and viscosity average molecular weight, respectively.

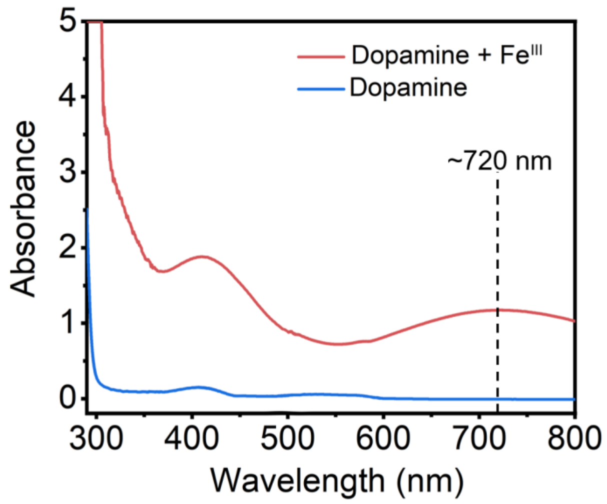

Supplementary Fig. 4 | UV-Vis spectra of solution containing dopamine and dopamine-Fe^{III} mixture (0.03 g sample dissolved in 100 mL solution).

Notes for Supplementary Fig. 4

An absorption peak around 720 nm was clearly observed for the dopamine-Fe^{III} mixture solution, suggesting that the coordination was mainly in the form of mono-catechol-Fe^{III}.

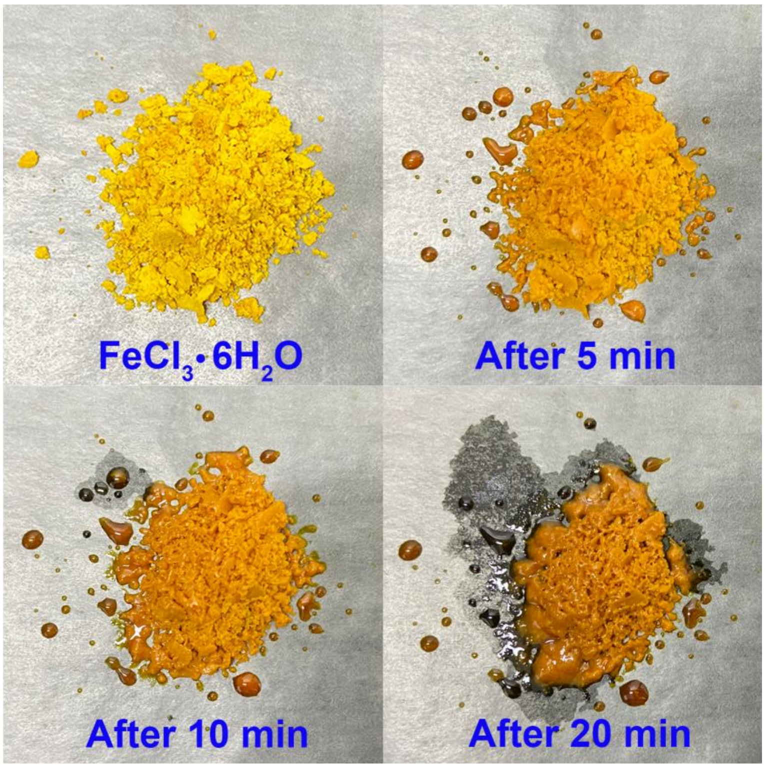

Supplementary Fig. 5 | Time course of the $FeCl_3$•$6H_2O$ deliquescence situation under air.

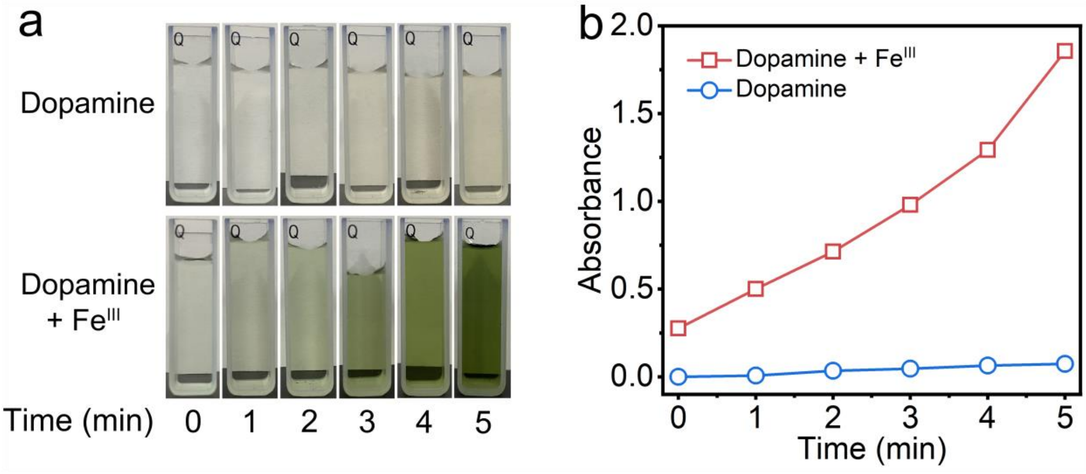

Supplementary Fig. 6 | Dopamine polymerization assisted by $FeCl_3•6H_2O$. a Photographs of dopamine and dopamine-Fe^{III} mixture solutions with different grinding times (0.03 g sample dissolved in 100 mL solution). **b** Time-dependence of absorbance at 420 nm for various dopamine solutions formed in different polymerization conditions.

Notes for Supplementary Fig. 6

The UV-Vis absorbance at 420 nm for various dopamine solutions was considered as the indicator of dopamine polymerization[1]. As a result, the color change and UV-Vis absorbance at 420 nm with reaction time conformed that the Fe^{III} could readily trigger the polymerization of dopamine.

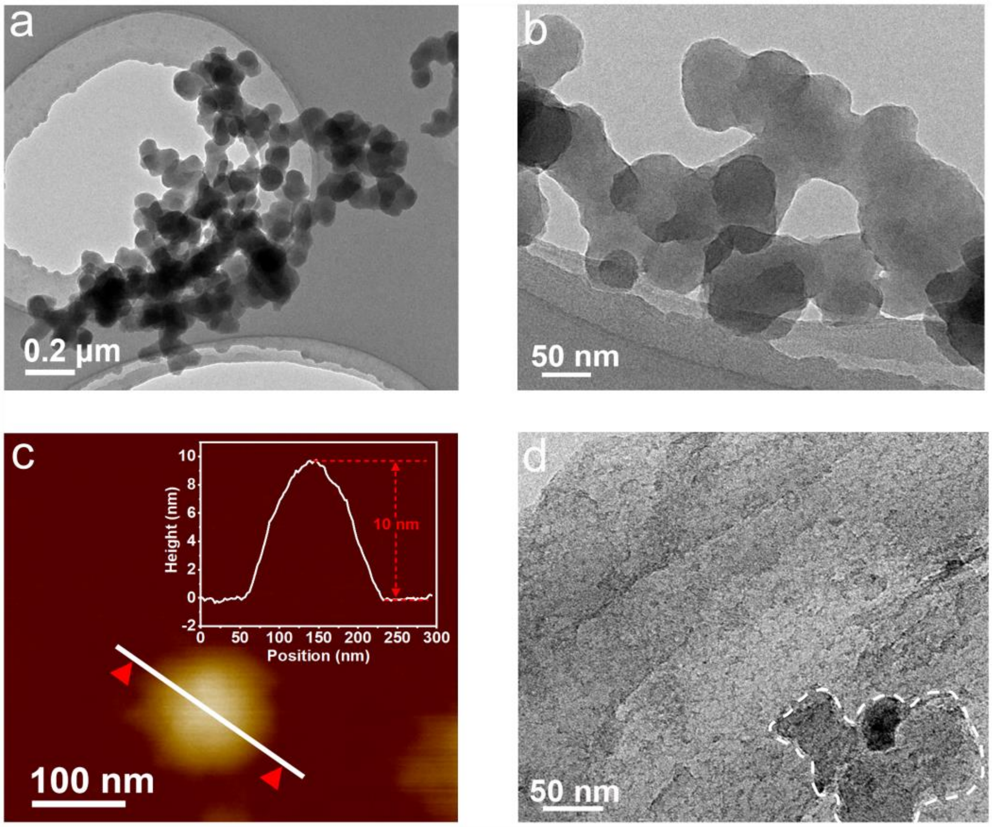

Supplementary Fig. 7 | Structural characterization of Fe-PDA complex and its calcined sample at 500 °C. a-b TEM images of Fe-PDA complex. **c** AFM image of Fe-PDA complex. **d** TEM image of Fe@N-C-500.

Notes for Supplementary Fig. 7

The Fe-PDA complex after grinding for 5 min was freeze-drying and then transferred for transmission electronic microscopy detection. From Supplementary Fig. 7a-c we could find that the Fe-PDA precursor presented the disc structure with the in-plane size from 50 to 200 nm and thickness of 10 nm, verifying the self-assembled layered structure of PDA. The TEM image of Fe@N-C-500 further confirmed that the 2D nanosheet of the catalyst was derived from the layered structure of PDA.

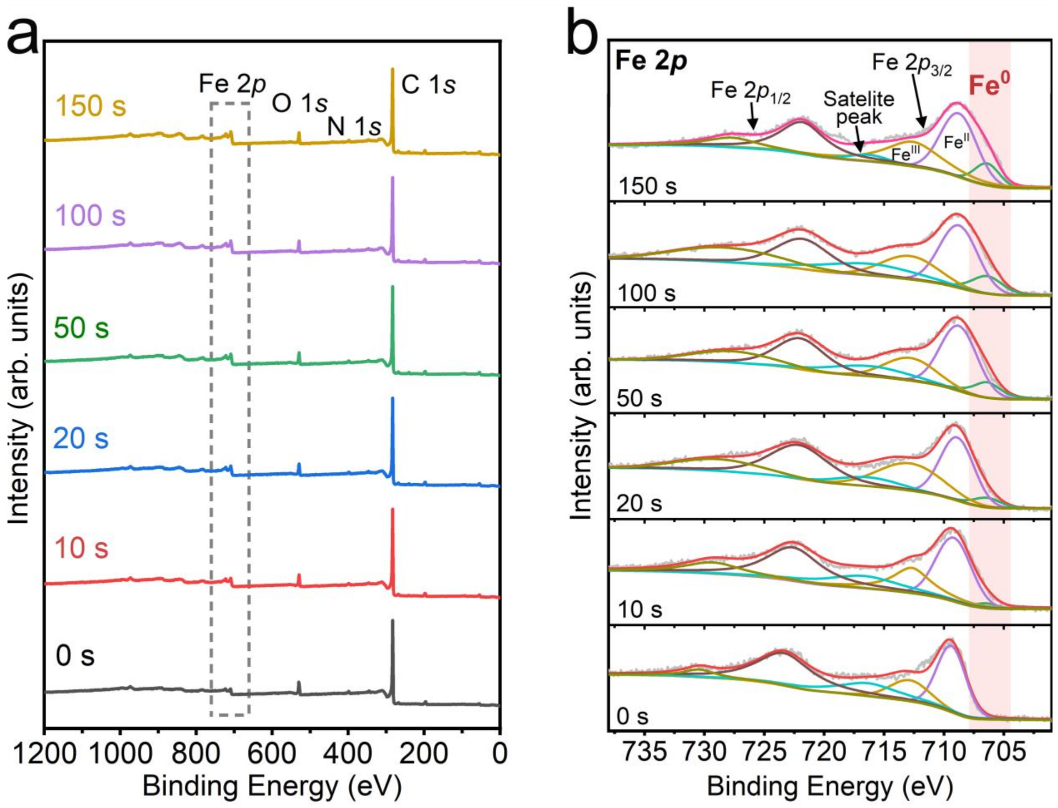

Supplementary Fig. 8 | XPS characterization of Fe@N-C-800 with different Ar etching times. a Survey spectra and **b** high-resolution spectra of Fe 2*p*.

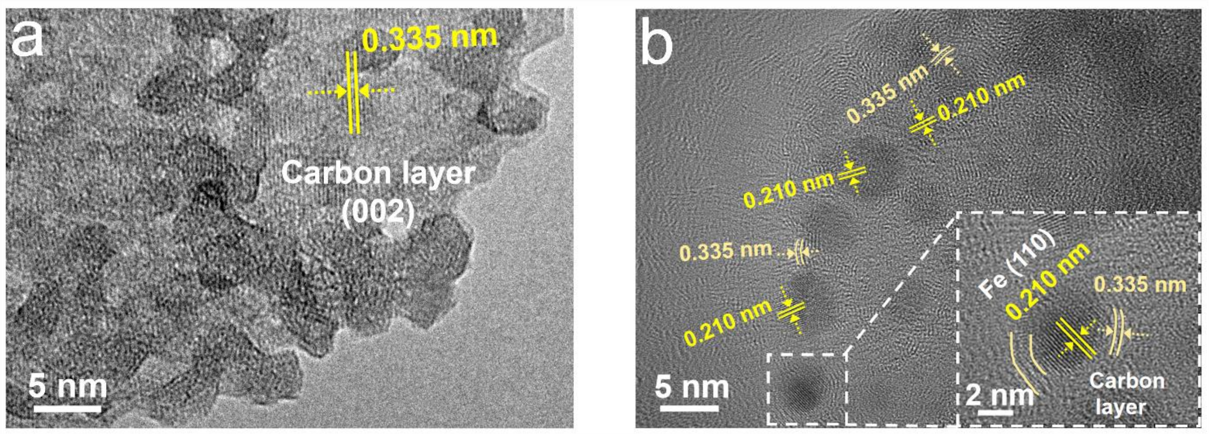

Supplementary Fig. 9 | HRTEM images of Fe@N-C-800 in different positions.

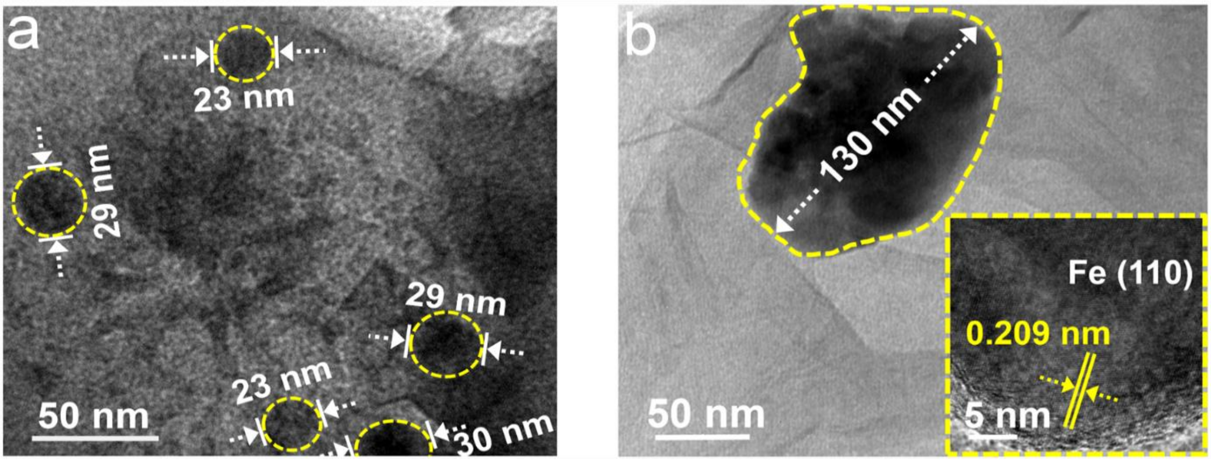

Supplementary Fig. 10 | TEM images of Fe@N-C synthesized at different conditions. a Fe(acac)$_3$ as a substitute for $FeCl_3$•$6H_2O$. **b** Fe-DA precursor under less oxidized condition.

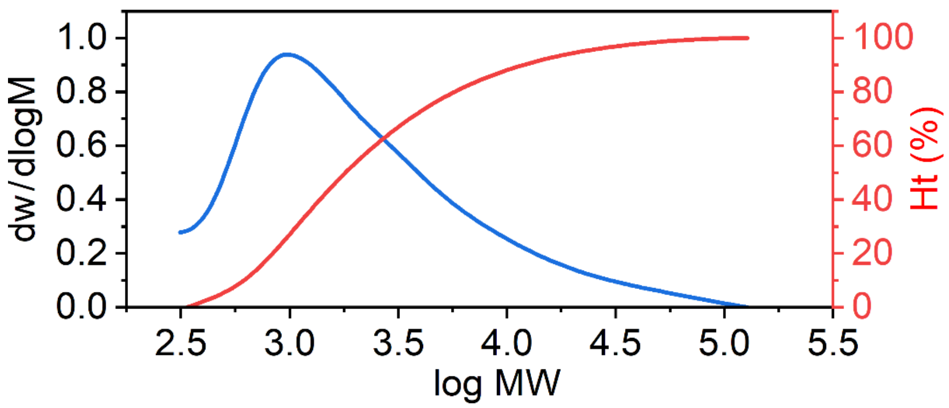

Supplementary Fig. 11 | GPC data of the Fe-DA complex under less oxidized condition (Fe^{II} and N_2 atmosphere).

MW Average

Peak No	Mp	Mn	Mw	Mz	Mz+1	Mv	PD
1	970	1291	**4799**	24835	52508	3619	3.71727

*Dimethyl sulfoxide (DMSO) was used as the solvent.

Notes for Supplementary Fig. 11

To demonstrate the effect of polymerization of DA on metal particle dispersion, we synthesized Fe@N-C (without polymerization) as a control sample. Here, we replaced Fe^{III} with less oxidized Fe^{II} as the metal precursor and the whole experimental place was filled with N_2 atmosphere to block the interference of O_2. The rest of the experimental steps were similar to the synthesis procedure of Fe@N-C-800.

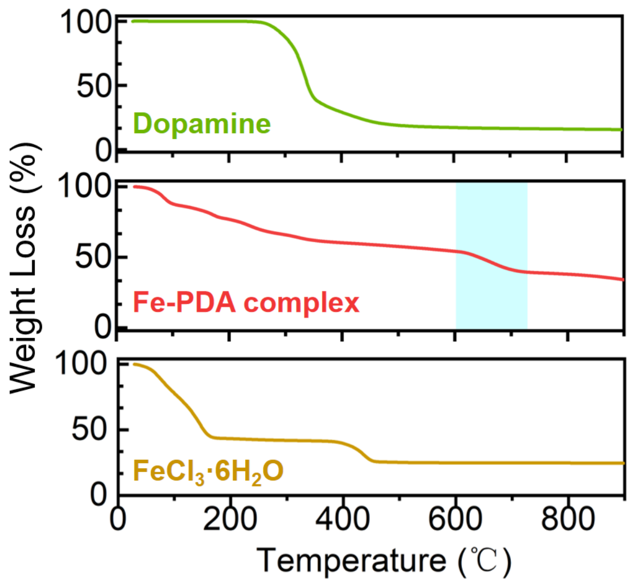

Supplementary Fig. 12 | Thermogravimetry analysis of various samples.

Notes for Supplementary Fig. 12

We simulated the changes of precursors during the pyrolysis process through thermogravimetry analyzer under N_2 atmosphere. The results showed that obvious weight loss of the Fe-PDA complex was detected during the calcination temperature range of 600-700 °C, indicating the evolution of Fe NPs at 700 °C, which was consistent with the following XRD results.

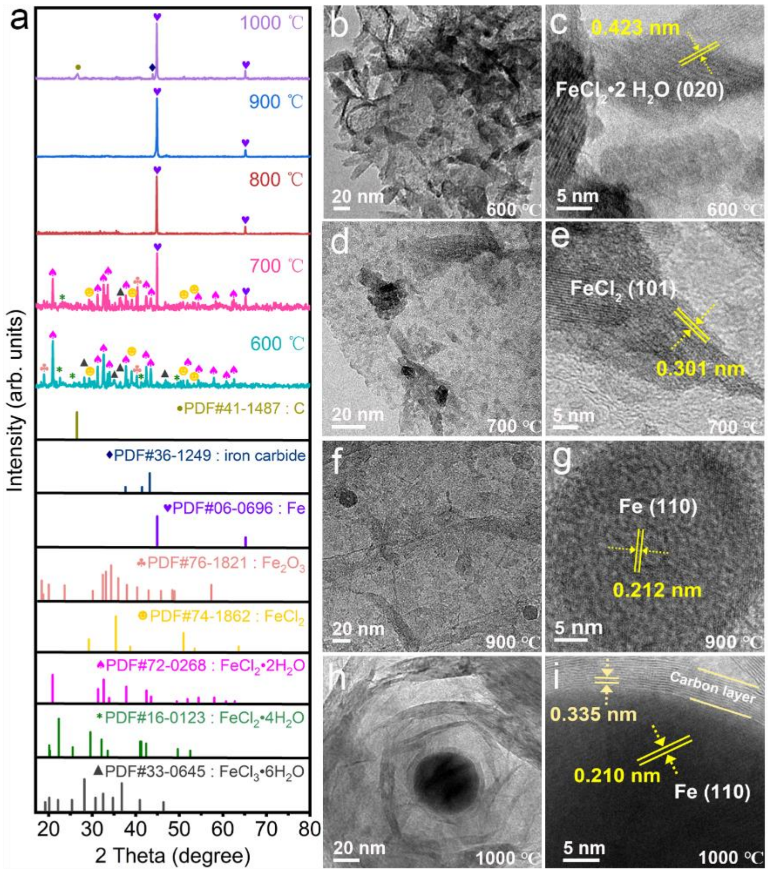

Supplementary Fig. 13 | Structural characterization of Fe@N-C-X synthesized under different temperatures. a XRD patterns. **b-i** TEM and HRTEM images.

Notes for Supplementary Fig. 13

As shown in Supplementary Fig. 13a, the sample calcined at 600 °C exhibited the obvious diffraction peaks of $FeCl_2•2H_2O$. With the increase of pyrolysis temperature, the diffraction peaks corresponding to the (110) and (200) crystal planes of Fe nanoparticles became more dominant. The sharp diffraction peaks in the XRD patterns might be ascribed to the existence of few large metal NPs in the catalysts. Nevertheless, considering that none of large metal NPs was observed in Fe@N-C-800 catalyst throughout the field of view (Fig. 1b, d) and the catalysts consisting of large metal NPs exhibited inferior catalytic abilities (Supplementary Fig. 22), the few large metal NPs could hardly affect the statistics of size distribution and the final Fenton catalytic ability of the Fe@N-C-800 in this work.

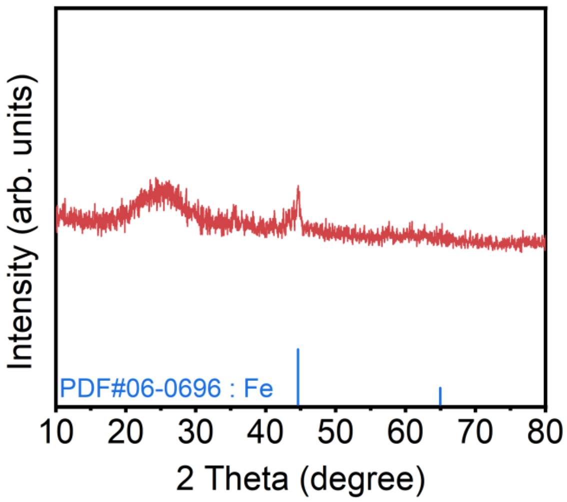

Supplementary Fig. 14 | XRD pattern of Fe@N-C-800 collected from the suspension.

Notes for Supplementary Fig. 14

Commonly, the samples for microscopic analysis are collected from the suspension after dispersing the pristine samples in solvent. Therefore, the XRD test for the catalyst collected from the suspension without the sediment was conducted. As shown in Supplementary Fig. 14, the diffraction peaks of Fe nanoparticles were greatly weakened in the catalyst collected from the suspension compared with that of pristine Fe@N-C-800 sample. The width at half maximum height of peaks located at 44.6° was 1.23°, thus the average size of Fe NPs was calculated to be 6.76 nm via the Scherrer equation, which was comparable with the results obtained from the TEM images.

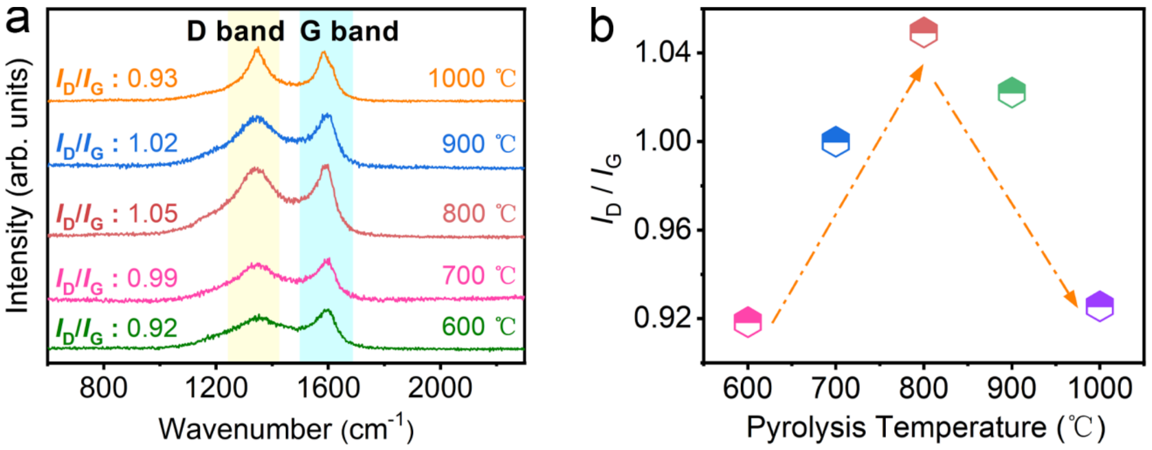

Supplementary Fig. 15 | Raman characterization of Fe@N-C-X synthesized under different temperatures. a Raman spectra. **b** Effect of pyrolysis temperatures on I_D/I_G values.

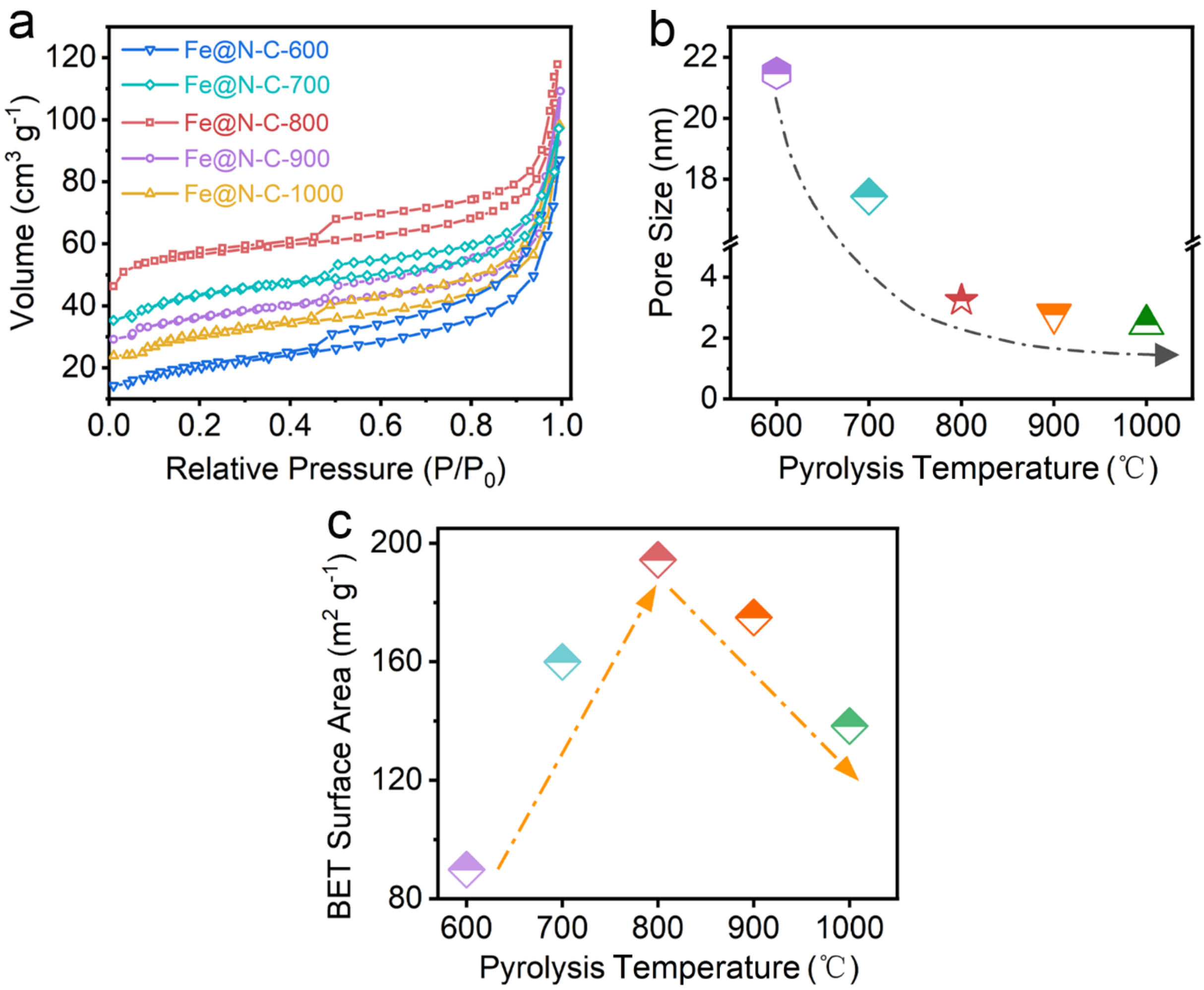

Supplementary Fig. 16 | N_2 adsorption/desorption measurements of Fe@N-C-X synthesized under different temperatures. a N_2 adsorption/desorption isotherms. Effects of pyrolysis temperatures on the **b** pore size and **c** specific surface area.

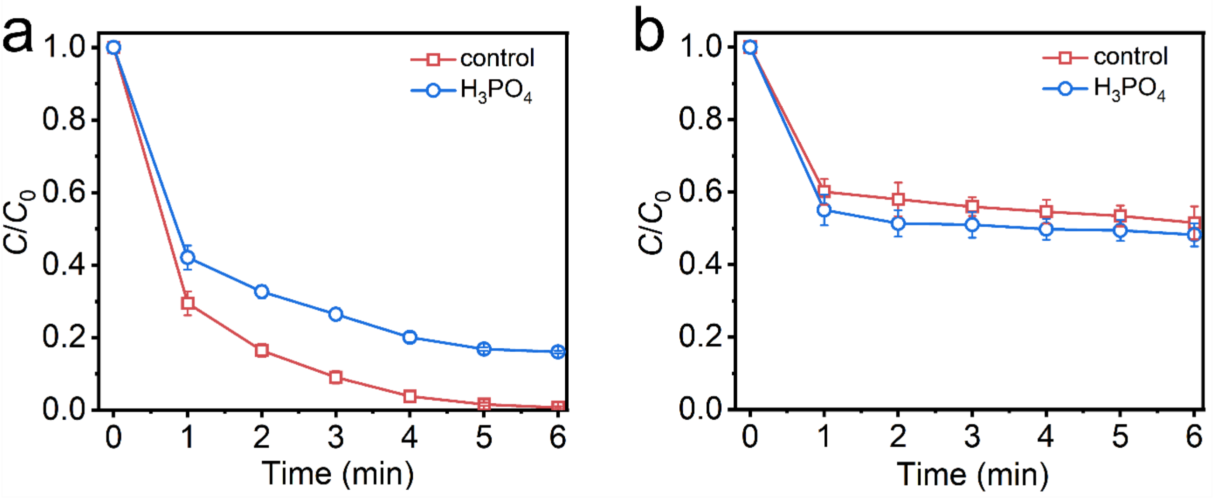

Supplementary Fig. 17 | Effect of H_3PO_4 on catalytic performance of different systems for SMX removal. **a** Fe@N-C-800/H_2O_2 system, **b** conventional Fenton system. Condition: [Fe@N-C-800] = 0.05 g L^{-1}, [Ferrous ions] = 2 mg L^{-1}, $[H_2O_2]_0$ = 1 × 10^{-3} M, $[SMX]_0$ = 10 × 10^{-6} M, $[H_3PO_4]$ = 0.1 M, T = 25 °C. The error bars in the figures represent the standard deviations.

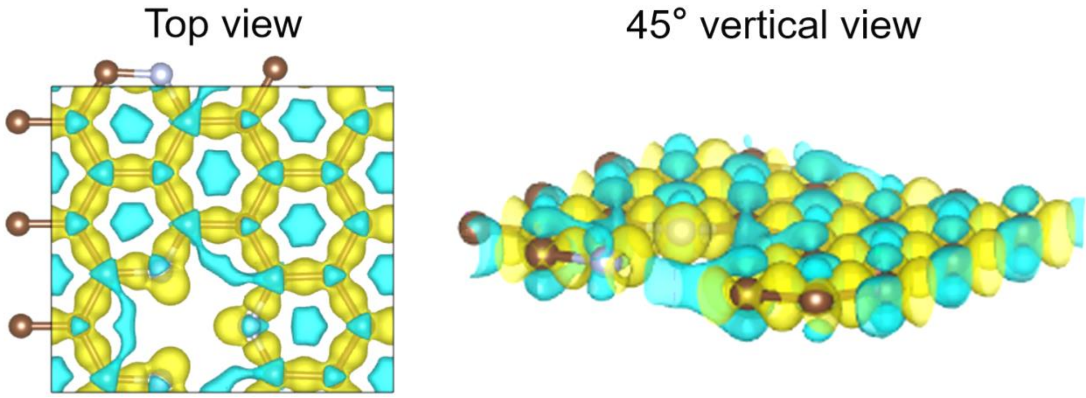

Supplementary Fig. 18 | The charge density differences in N-C. Yellow and cyan regions represent electron accumulation and depletion, respectively. The brown and silver respectively denote C and N atoms.

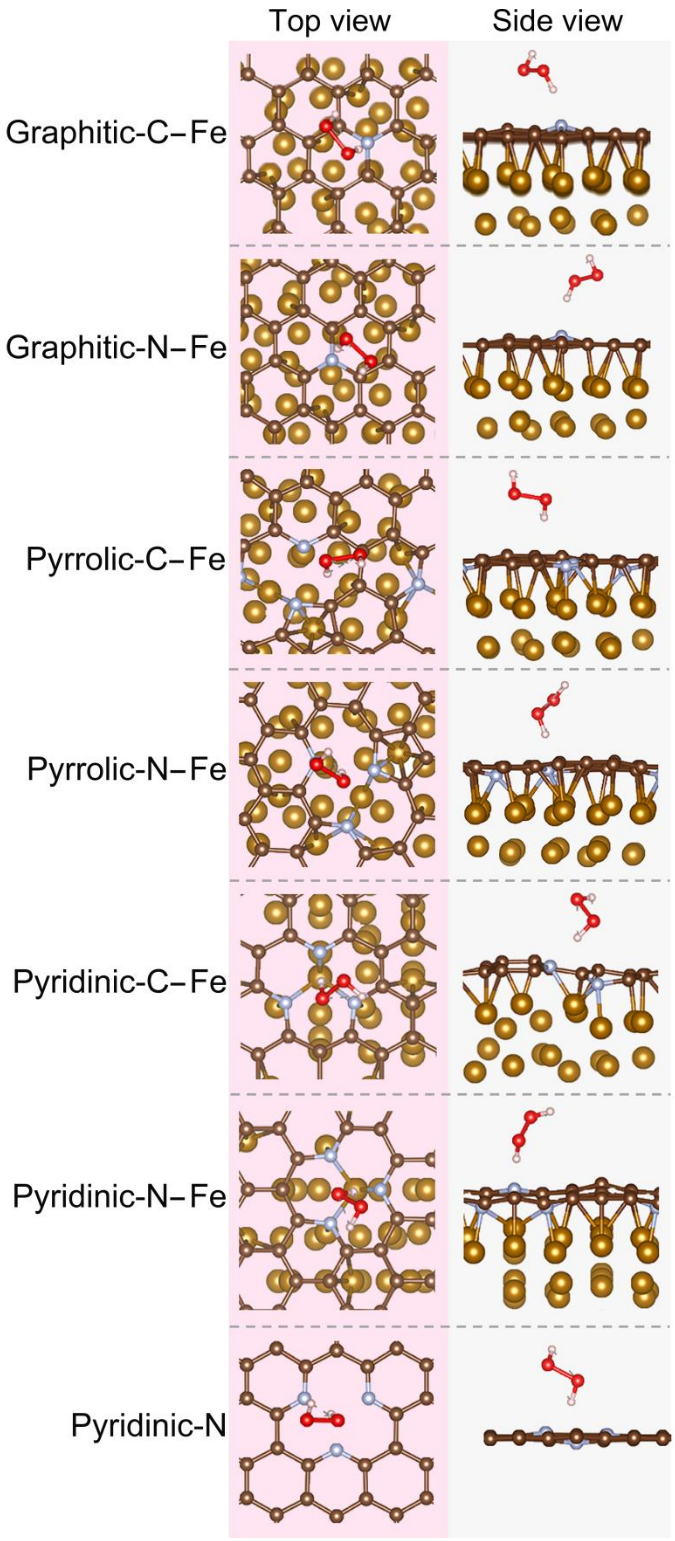

Supplementary Fig. 19 | Theoretical calculation models of different active sites from multiple perspectives for the Fe@N-C and N-C materials. The golden, silver, brown, red and white respectively denote Fe, N, C, O and H atoms.

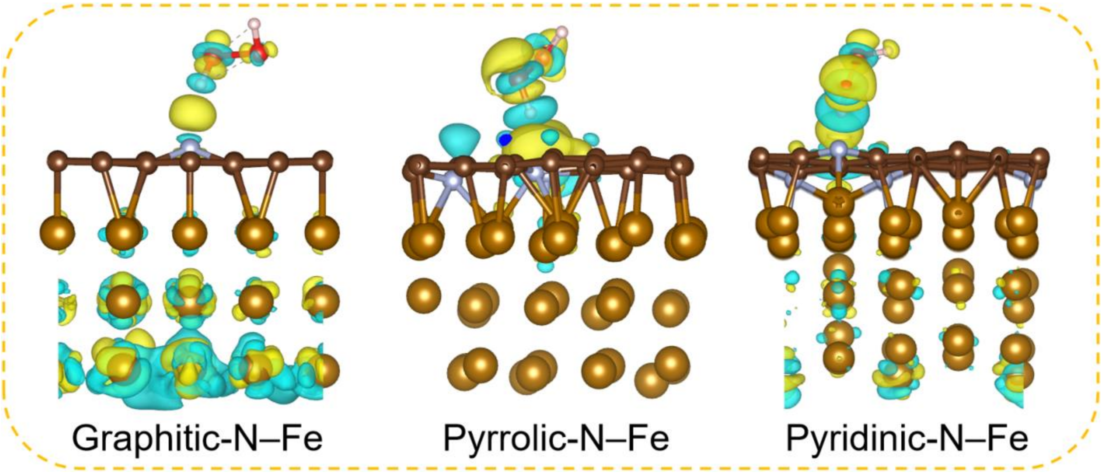

Supplementary Fig. 20 | The charge density differences of the adsorption of H_2O_2 on different sites. The golden, silver, brown, red and white respectively denote Fe, N, C, O and H atoms. Yellow and cyan regions represent electron accumulation and depletion, respectively.

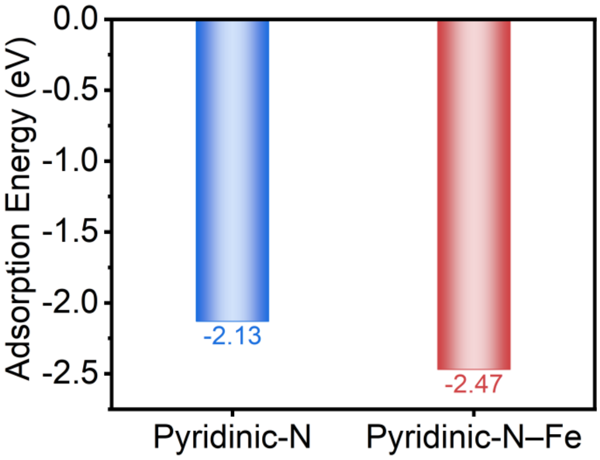

Supplementary Fig. 21 | Adsorption energy of pyridinic-N and pyridinic-N–Fe sites for •OH.

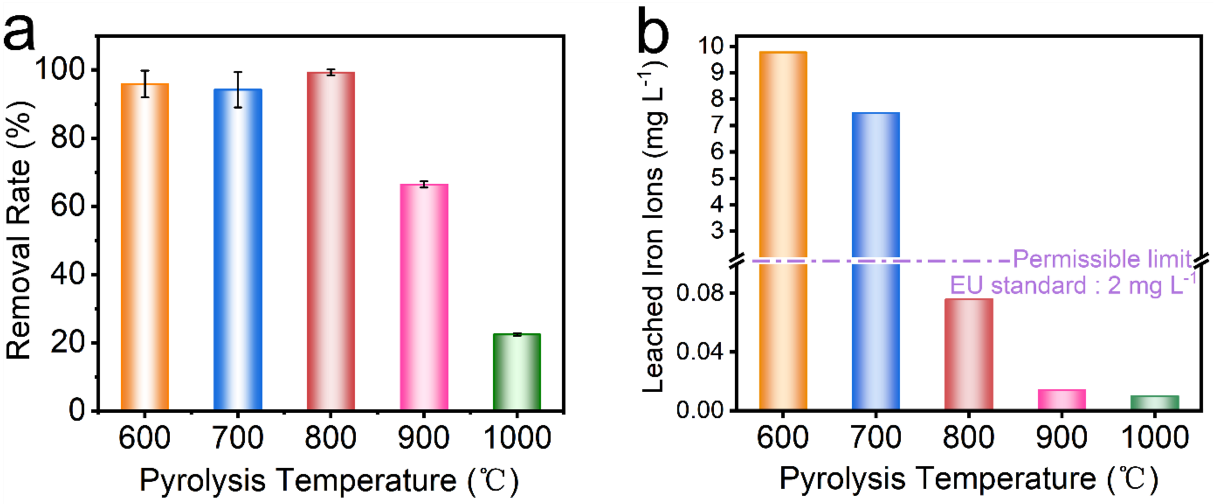

Supplementary Fig. 22 | Fenton reaction performance of Fe@N-C-X prepared at different temperatures. **a** Contaminant removal performance. **b** Leached iron ions during reaction. Condition: [Catalyst] = 50 mg L^{-1}, $[H_2O_2]_0$ = 1 × 10^{-3} M, $[SMX]_0$ = 10 × 10^{-6} M, initial pH = 6.2, T = 25 °C. The error bars in the figures represent the standard deviations.

Notes for Supplementary Fig. 22

Considering the important roles of synthetic temperatures on the catalytic performance, the effects of pyrolysis temperature with respect to the target pollutant removal efficiency was explored. We found that the precursors by pyrolysis at 800 °C could achieve the superior heterogenous catalytic performance (Supplementary Fig. 22a). Supplementary Fig. 22b displayed the concentration of leached iron ions in Fe@N-C-X during catalytic reaction. The reasons for high iron leaching of Fe@N-C-600 and Fe@N-C-700 was attributed to the presence of water-soluble $FeCl_2$•$2H_2O$ and $FeCl_2$. By contrast, encapsulating metallic Fe into carbon framework was almost achieved as thermal treatment rose to 800 °C, thus inhibiting the leaching of iron ions during catalytic reaction.

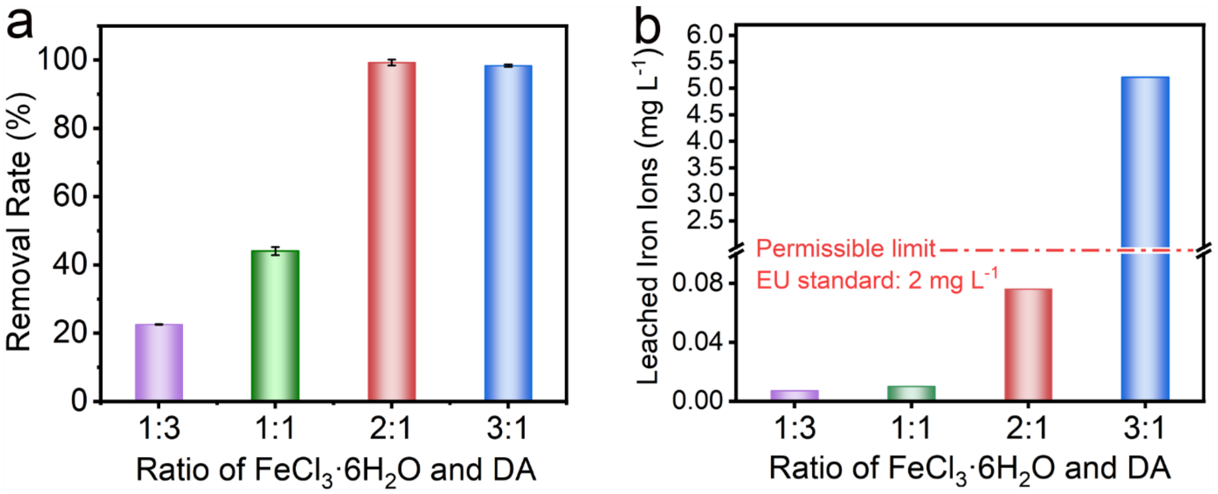

Supplementary Fig. 23 | Fenton reaction performance of Fe@N-C-800 prepared with different ratios of $FeCl_3$•$6H_2O$ and DA. a Contaminant removal performance. **b** Leached iron ions during reaction. Condition: [Catalyst] = 50 mg L^{-1}, $[H_2O_2]_0 = 1 \times 10^{-3}$ M, $[SMX]_0 = 10 \times 10^{-6}$ M, initial pH = 6.2, T = 25 °C. The error bars in the figures represent the standard deviations.

Notes for Supplementary Fig. 23

The influence of $FeCl_3$•$6H_2O$/DA ratio (from 1:3 to 3:1) on the Fe@N-C-800 catalytic performance was also investigated. We found that the precursor component of $FeCl_3$•$6H_2O$/DA with molar ratio of 2:1 could achieve the superior heterogenous catalytic performance (Supplementary Fig. 23a). It should be noted that the leached ions (Supplementary Fig. 23b) also increased along with the $FeCl_3$•$6H_2O$/DA molar ratios due to the inadequate coordination between DA and excessive Fe^{III}, and thus only the ratio of 2:1 could achieve the splendid heterogenous catalytic performance.

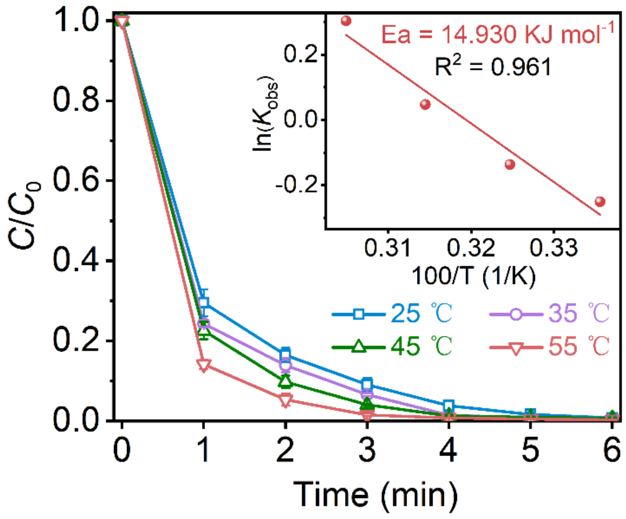

Supplementary Fig. 24 | SMX removal under different reaction temperatures, and the inset shows the Arrhenius plot of the Fe@N-C-800/H_2O_2 system. Condition: $[SMX]_0 = 10 \times 10^{-6}$ M, $[H_2O_2]_0 = 1 \times 10^{-3}$ M, [Fe@N-C-800] = 50 mg L^{-1}, initial pH = 6.2. The error bars in the figures represent the standard deviations.

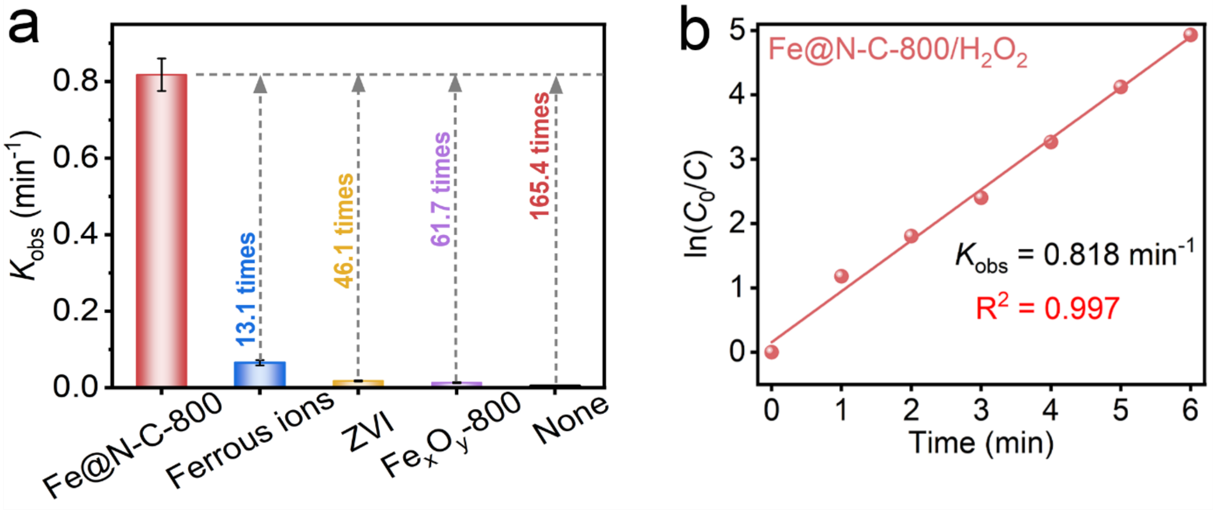

Supplementary Fig. 25 | SMX removal performance of different systems. a K_{obs} values in various catalyst induced H_2O_2-based systems. **b** Kinetics analysis of the Fe@N-C-800/H_2O_2 system. Condition: [Fe@N-C-800] = [ZVI] = [Fe_xO_y-800] = 50 mg L^{-1}, [Ferrous ions] = 2 mg L^{-1}, $[H_2O_2]_0 = 1 \times 10^{-3}$ M, $[SMX]_0 = 10 \times 10^{-6}$ M, initial pH = 6.2, T = 25 °C. The error bars in the figures represent the standard deviations.

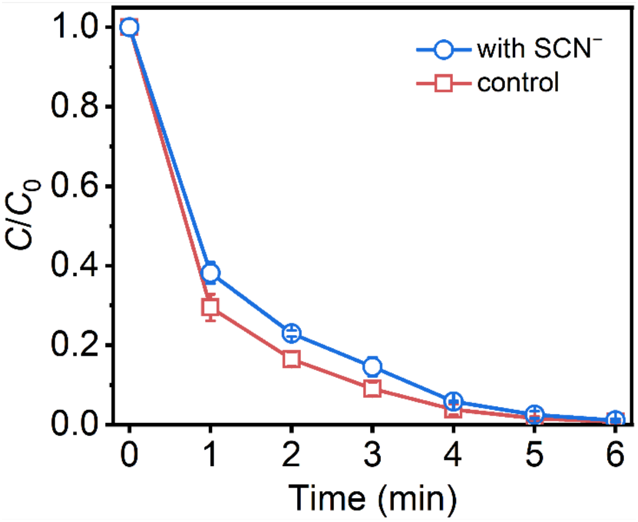

Supplementary Fig. 26 | The SMX degradation performance of the Fe@N-C-800 before and after the addition of KSCN. Condition: [Fe@N-C-800] = 50 mg L^{-1}, $[H_2O_2]_0 = 1 \times 10^{-3}$ M, $[SMX]_0 = 10 \times 10^{-6}$ M, [KSCN] = 10×10^{-3} M, initial pH = 6.2, T = 25 °C. The error bars in the figures represent the standard deviations.

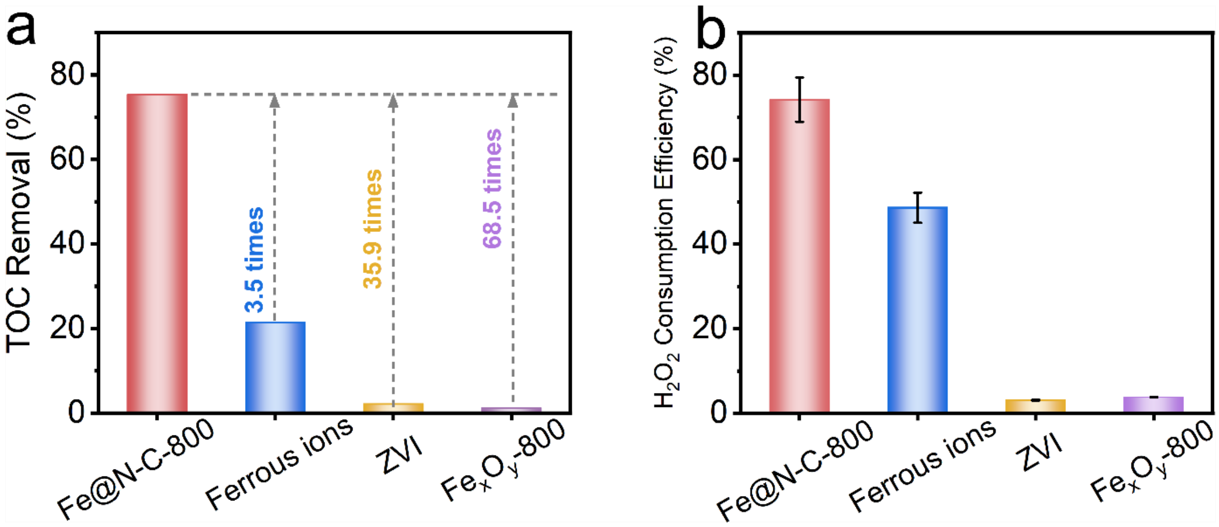

Supplementary Fig. 27 | TOC removal and the corresponding H_2O_2 consumption efficiency of different catalysts. a TOC removal performance. **b** H_2O_2 consumption efficiency. Condition: [Fe@N-C-800] = [ZVI] = [Fe_xO_y-800] = 50 mg L^{-1}, [Ferrous ions] = 2 mg L^{-1}, $[H_2O_2]_0 = 2 \times 10^{-3}$ M, $[SMX]_0 = 50 \times 10^{-6}$ M, initial pH = 6.2, T = 25 °C, reaction time = 30 min. The error bars in the figures represent the standard deviations.

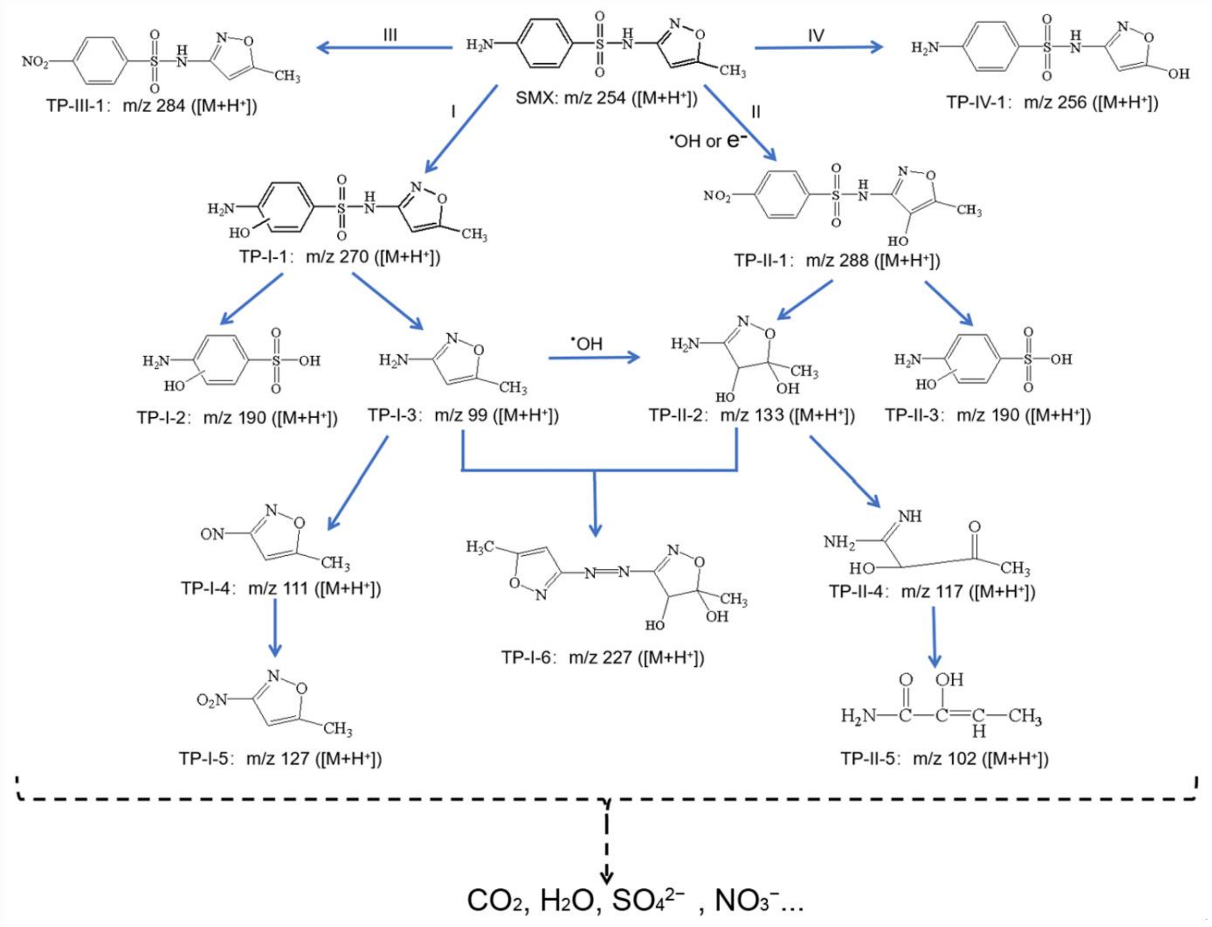

Supplementary Fig. 28 | SMX degradation pathway in the Fe@N-C-800/H_2O_2 system.

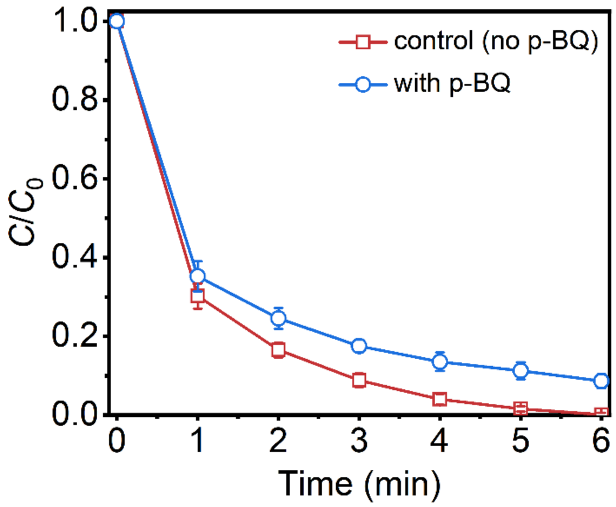

Supplementary Fig. 29 | Effect of p-BQ on pollutant removal in the Fe@N-C-800/H_2O_2 system. Condition: [Fe@N-C-800] = 50 mg L^{-1}, $[H_2O_2]_0 = 1 \times 10^{-3}$ M, $[SMX]_0 = 10 \times 10^{-6}$ M, [p-BQ] = 10×10^{-3} M, initial pH = 6.2, T = 25 °C. The error bars in the figures represent the standard deviations.

*p-BQ is a typical scavenger of $^{\bullet}O_2H$.

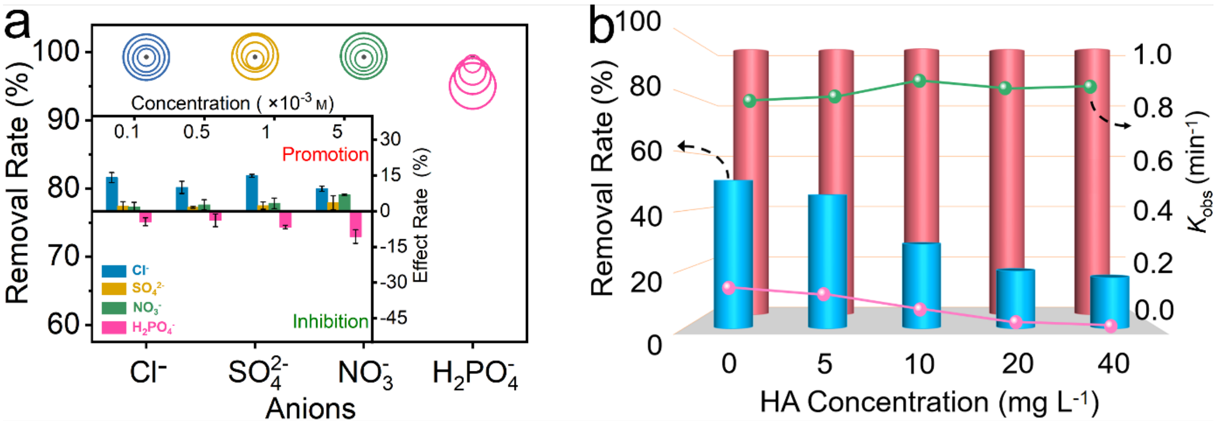

Supplementary Fig. 30 | Effects of anions and HA on SMX removal. a Effect of anions on SMX removal in the Fe@N-C-800/H_2O_2 system, and the inset displays the variation of K_{obs} under different concentrations of anions. The size of circles is positively related to the concentration of anions, and the dots mean the original system in the absence of anions. **b** Effect of HA on SMX removal in the Fe@N-C-800/H_2O_2 system (red column) and conventional Fenton system (blue column). Condition: [Fe@N-C-800] = 50 mg L^{-1}, [Ferrous ions] = 2 mg L^{-1}, $[H_2O_2]_0 = 1 \times 10^{-3}$ M, $[SMX]_0 = 10 \times 10^{-6}$ M, T = 25 °C. The error bars in the figures represent the standard deviations.

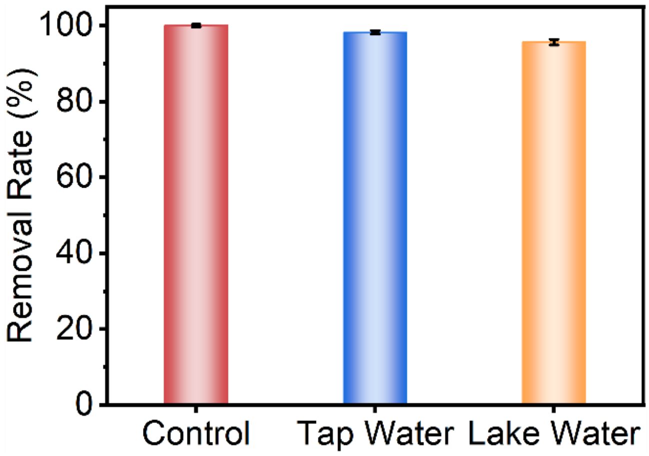

Supplementary Fig. 31 | Effect of water matrix on the removal of SMX in the Fe@N-C-800/H_2O_2 system. Condition: [Fe@N-C-800] = 50 mg L^{-1}, $[SMX]_0 = 10 \times 10^{-6}$ M, $[H_2O_2]_0 = 1 \times 10^{-3}$ M, T = 25 °C. The error bars in the figures represent the standard deviations.

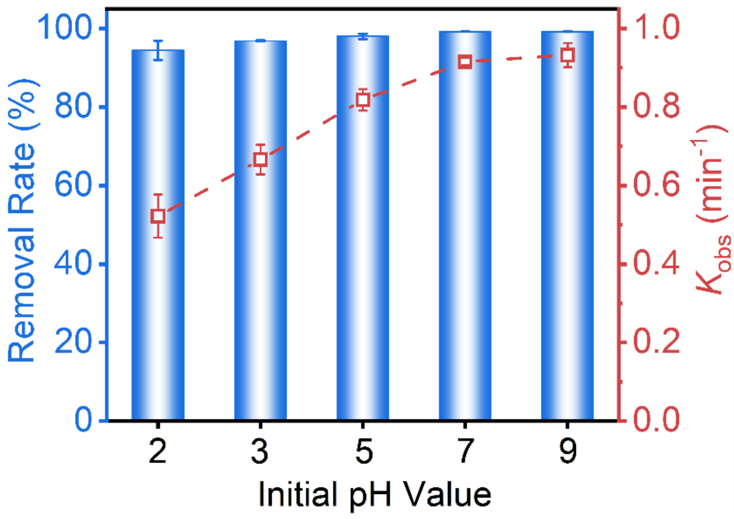

Supplementary Fig. 32 | Effect of initial pH on SMX removal and K_{obs} in the Fe@N-C-800/H_2O_2 system. Condition: [Fe@N-C-800] = 50 mg L^{-1}, $[H_2O_2]_0 = 1 \times 10^{-3}$ M, $[SMX]_0 = 10 \times 10^{-6}$ M, T = 25 °C. The error bars in the figures represent the standard deviations.

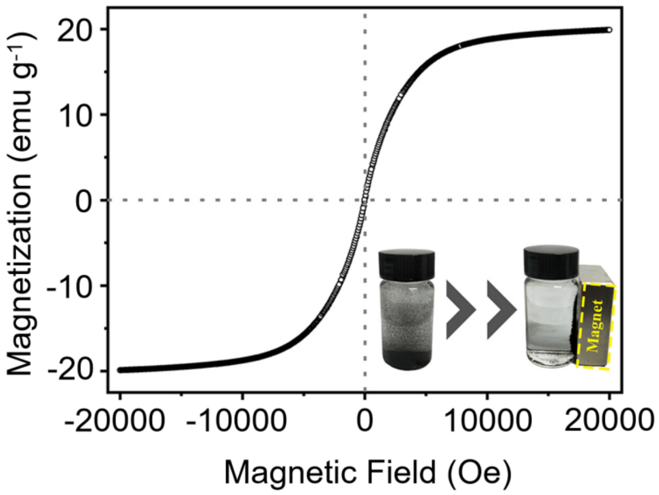

Supplementary Fig. 33 | Room temperature hysteresis loop of Fe@N-C-800.

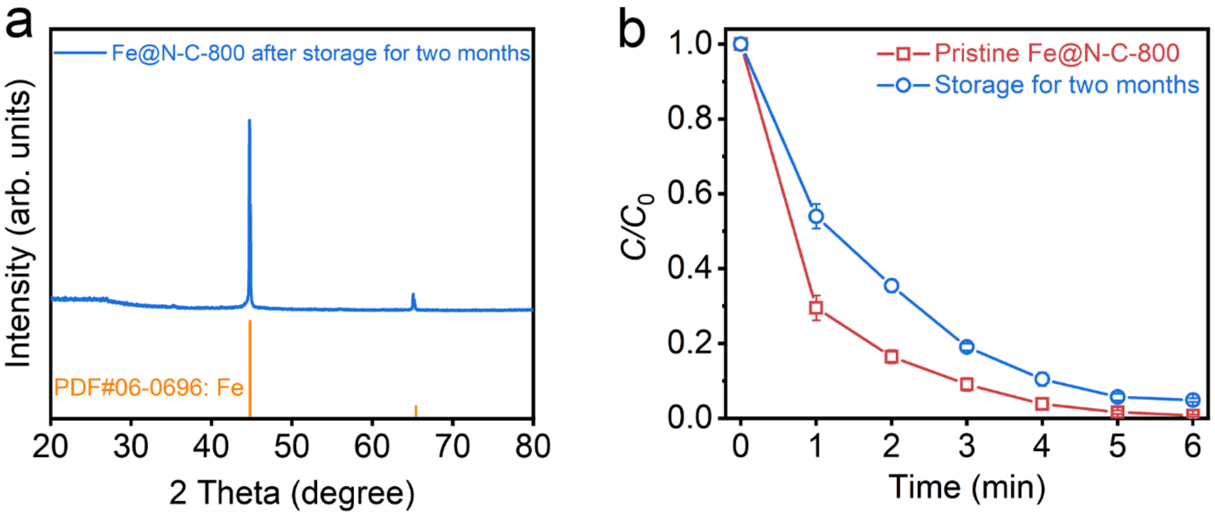

Supplementary Fig. 34 | Structural characterization and catalytic performance of Fe@N-C-800 after storage for two months. a XRD pattern of Fe@N-C-800 after storage for two months. **b** Comparison of the catalytic performance for SMX removal between pristine Fe@N-C-800 and the sample after storage for two months. Condition: [Fe@N-C-800] = 50 mg L^{-1}, $[H_2O_2]_0 = 1 \times 10^{-3}$ M, $[SMX]_0 = 10 \times 10^{-6}$ M, initial pH = 6.2, T = 25 °C. The error bars in the figures represent the standard deviations.

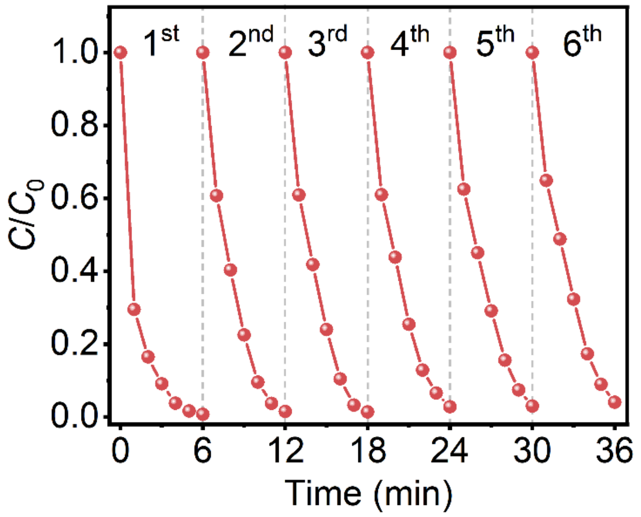

Supplementary Fig. 35 | Cyclic degradation of SMX by the recycled Fe@N-C-800. Condition: $[SMX]_0$ = 10 × 10^{-6} M, $[H_2O_2]$ = 1 × 10^{-3} M, T = 25 °C.

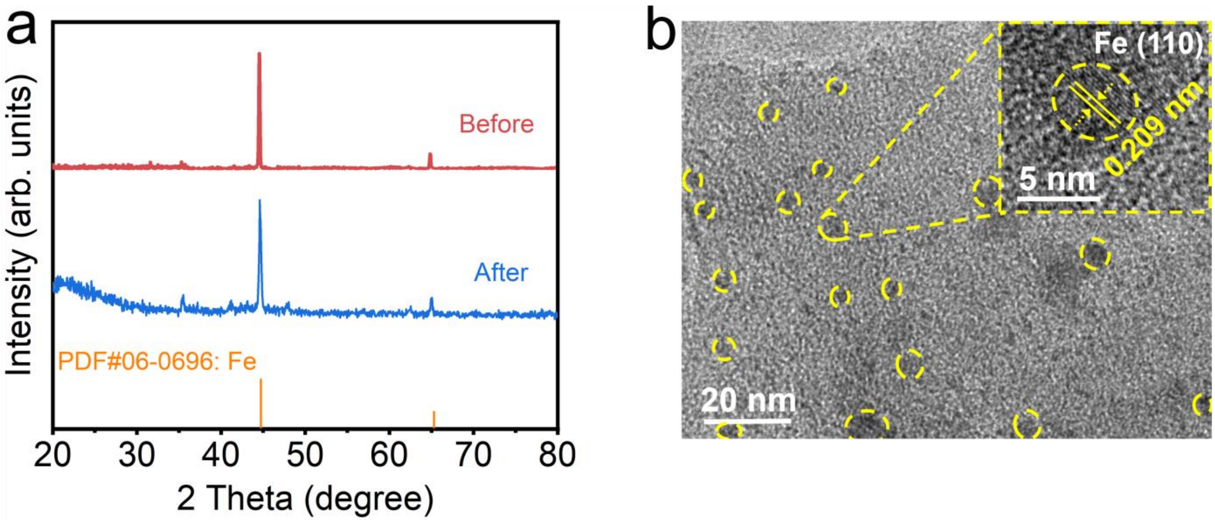

Supplementary Fig. 36 | Structural characterization of Fe@N-C-800 after reaction. a XRD patterns of Fe@N-C-800 before and after reaction. **b** TEM image of Fe@N-C-800 after reaction.

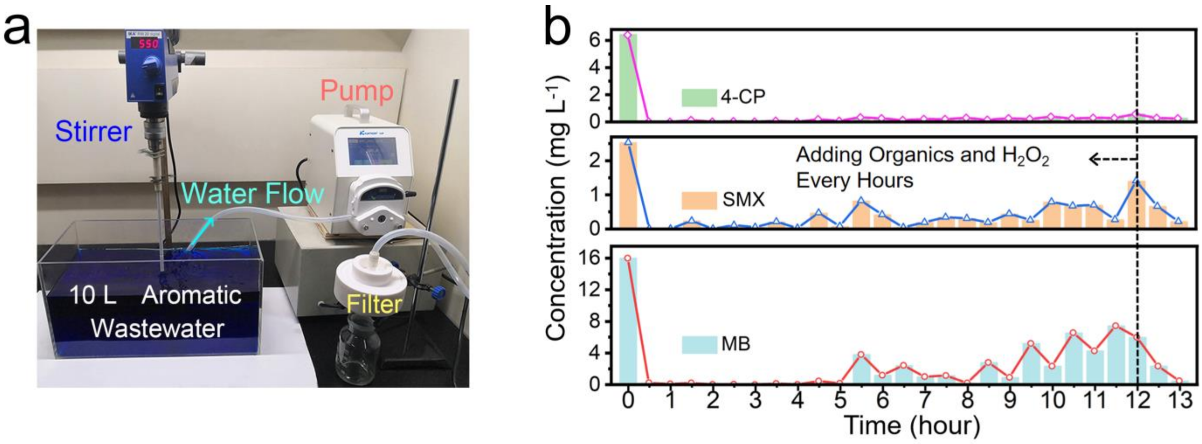

Supplementary Fig. 37 | The pilot-scale application of Fe@N-C-800 in wastewater treatment. a Photograph of the experimental device. **b** 13 hours continuous degradation of 10 L aromatic organics (total added [SMX] = 30.36 mg L^{-1}, [4-CP] = 77.16 mg L^{-1} and [MB] = 192 mg L^{-1}). Condition: [Fe@N-C-800] = 50 mg L^{-1}, initial pH = 6.2, T = 25 °C.

Notes for Supplementary Fig. 37

We conducted a pilot-scale setup equipped with a transparent acrylic plexiglass tank, peristaltic pump, electric motor stirrer and miniaturized Teflon filter holder for the continuous degradation of aromatic organics (Supplementary Fig. 37a), where a certain concentration of H_2O_2 and aromatic organics were injected at a given time interval of 1 hour. Specifically, the aromatic wastewater containing SMX, 4-CP and MB was prepared with 10 L tap water, and the initial concentration of SMX, 4-CP and MB were 10×10^{-6} M, 50×10^{-6} M and 50×10^{-6} M, respectively, at time = 0 hour. For treating the aromatic wastewater, 0.5 g Fe@N-C-800 was added into the wastewater at time = 0 hour, 2 mL H_2O_2 (30 wt%) was added every hours, additional SMX, 4-CP and MB with the same content as the initial values were added at every hours from time = 1 to 11 hour. To our delight, over 97% removal efficiency of three aromatic organics (SMX, 4-CP and MB) could be achieved after 13 hours continuous reaction (Supplementary Fig. 37b), demonstrating the feasibility of Fe@N-C-800 in treating large amount of wastewater.

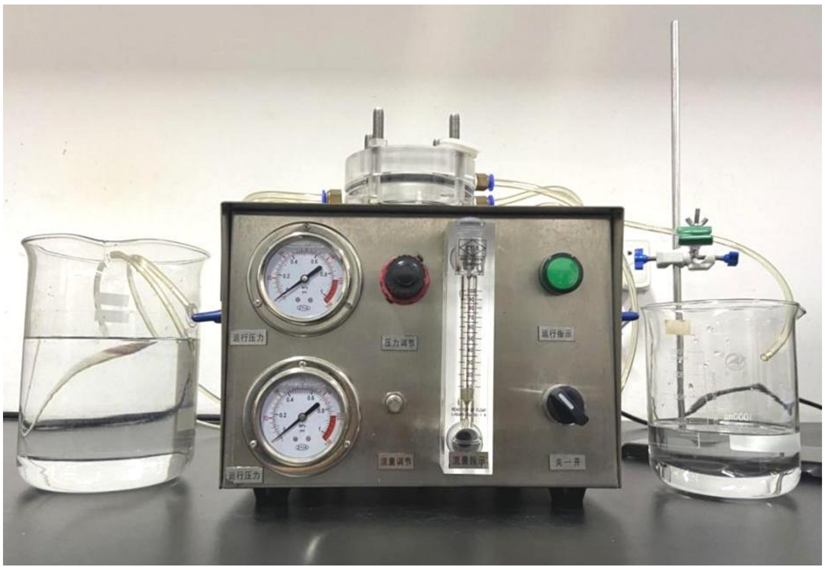

Supplementary Fig. 38 | Image of a cross-flow filtration equipment.

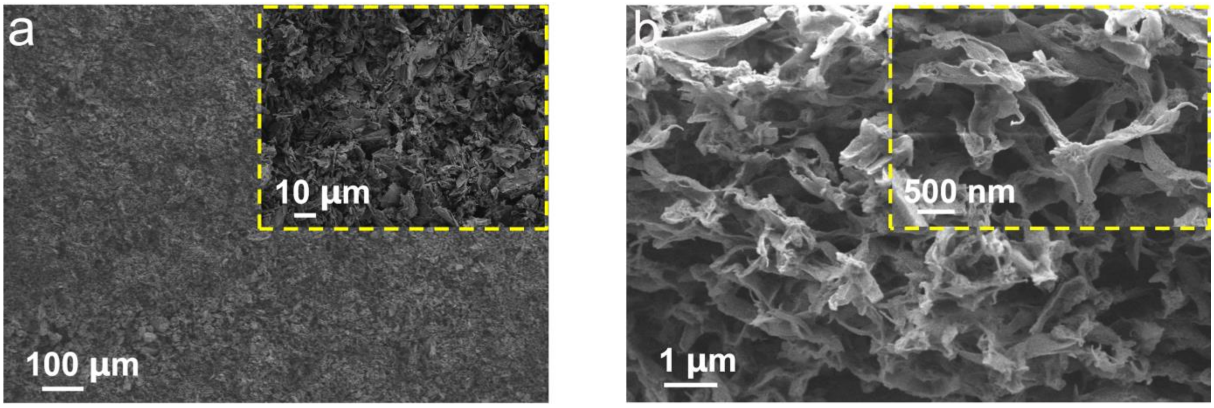

Supplementary Fig. 39 | SEM images of Fe@N-C-800/PVDF membrane. a The top view and **b** the cross-sectional view.

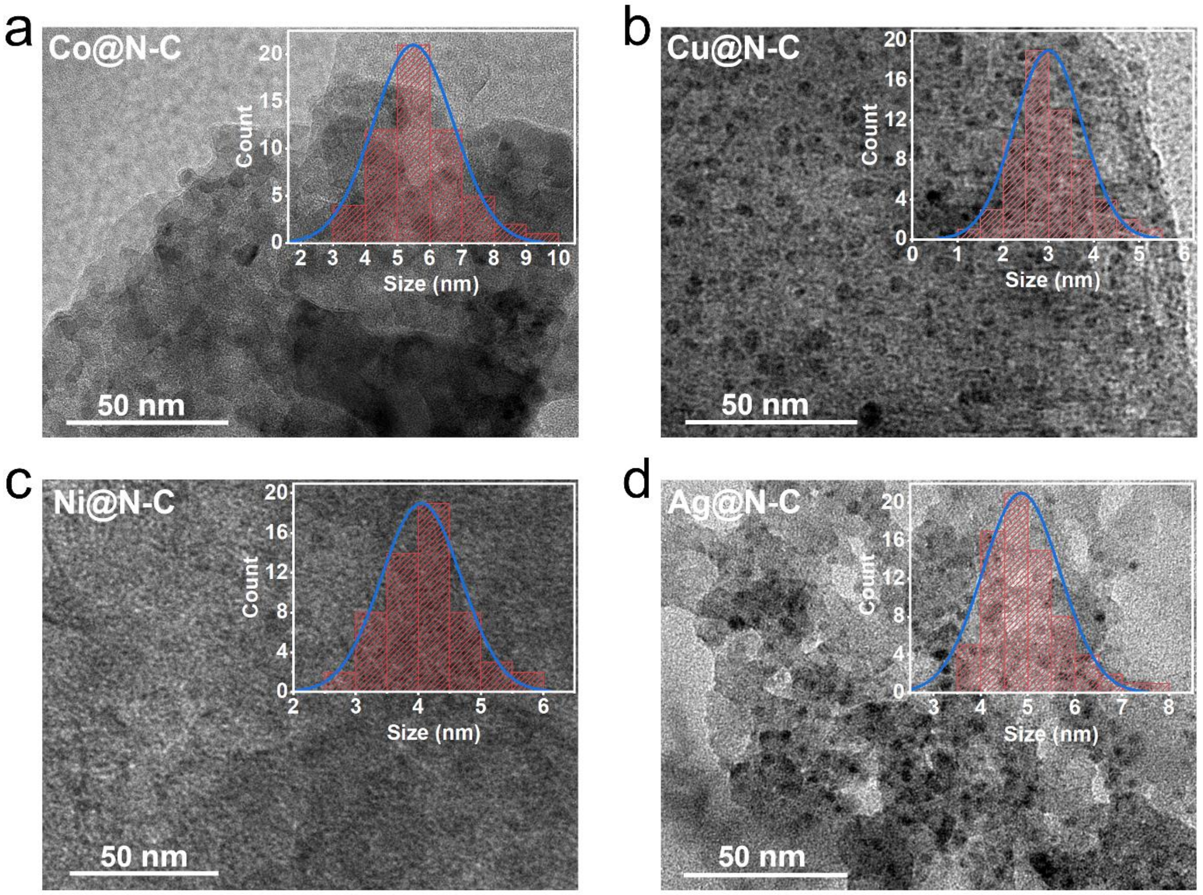

Supplementary Fig. 40 | TEM images of different M@N-C materials. a Co@N-C, **b** Cu@N-C, **c** Ni@N-C, **d** Ag@N-C (insets: size distribution histograms of the different M@N-C materials).

Notes for Supplementary Fig. 40

The TEM images demonstrated the highly dispersed metallic components on the carbon nanosheets, while the XRD patterns of the M@N-C materials exhibited the sharp diffraction peaks. This phenomenon could be attributed to the existence of few large metal NPs, which was consistent with the Fe@N-C materials and was elucidated in Supplementary Fig. 13-14.

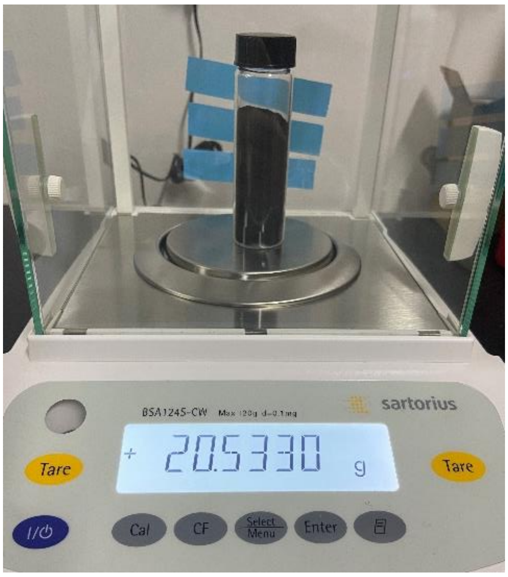

Supplementary Fig. 41 | Picture of the catalysts synthesized at a time.

Supplementary Tables

Supplementary Table 1 | Mössbauer hyperfine parameters derived from Mössbauer spectra of Fe@N-C-800 before and after reaction.

Sample	Assignment	Hyperfine field (KOe)	Isomer shift (mm s^{-1})	Quadrupole splitting (mm s^{-1})	Spectral area (%)	Component
Pristine Fe@N-C-800	D1	—	0.36	0.77	12.3	Fe^{III}
	Sext1	330.65	0	-0.01	64.0	α-Fe
	Sext2	486.84	0.40	0.12	1.2	Fe_3O_4
	Sext3	469.22	0.97	0.49	6.0	Fe_3O_4
	Sext4	207.12	0.23	-0.05	16.5	iron carbide
Used Fe@N-C-800	D1	—	0.36	0.72	27.1	Fe^{III}
	Sext1	330.65	0	-0.01	51.3	α-Fe
	Sext2	486.84	0.40	0.12	1.7	Fe_3O_4
	Sext3	469.22	0.97	0.49	4.1	Fe_3O_4
	Sext4	207.12	0.23	-0.05	15.8	iron carbide

Supplementary Table 2 | The BET surface area and pore size for Fe@N-C-X.

Sample	BET surface area (m^2 g^{-1})	Average pore size (nm)
Fe@N-C-600	89.82	21.47
Fe@N-C-700	159.96	17.43
Fe@N-C-800	194.42	3.24
Fe@N-C-900	174.93	2.78
Fe@N-C-1000	138.30	2.45

Supplementary Table 3 | Parameters of H_2O_2 adsorbed on the different sites.

Site	Adsorption energy (ΔE) of H_2O_2 (eV)
Graphitic-C–Fe	-0.14
Graphitic-N–Fe	-0.16
Pyrrolic-C–Fe	-0.38
Pyrrolic-N–Fe	-0.40
Pyridinic-C–Fe	-0.47
Pyridinic-N–Fe	-0.67
Pyridinic-N	-0.52

*ΔE is the H_2O_2 adsorption energy and the negative adsorption energy indicates that the total energy decreases when H_2O_2 is adsorbed.

Supplementary Table 4 | Calculated empirical formula and experimental m/z values of the protonated molecular ions of detected intermediates and their main fragments.

Molecular formula	Product name	Theoretical mass (m/z)	Measured mass (m/z)	Proposed structure
$C_{10}H_{11}N_3O_3S$	SMX	254.0599	254.0608	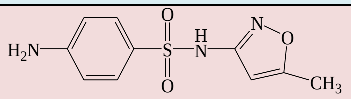
$C_{10}H_{11}N_3O_4S$	TP-I-1	270.0549	270.0567	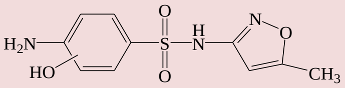
$C_6H_7NO_4S$	TP-I-2<br>TP-II-3	190.0174	190.0167	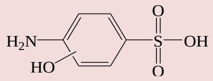
$C_4H_6N_2O$	TP-I-3	99.0558	99.0558	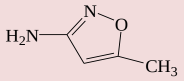
$C_4H_4N_2O_2$	TP-I-4	111.0195	111.0212	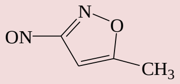
$C_4H_4N_2O_3$	TP-I-5	127.0144	127.0157	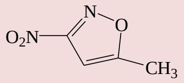
$C_8H_{10}N_4O_4$	TP-I-6	227.0780	227.0782	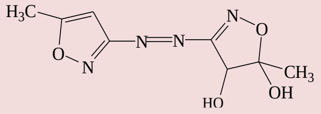
$C_{10}H_{13}N_3O_5S$	TP-II-1	288.0654	288.0634	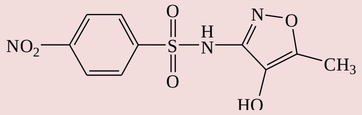
$C_4H_8N_2O_3$	TP-II-2	133.0613	133.0617	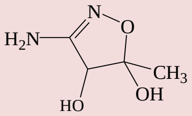
$C_4H_8N_2O_2$	TP-II-4	117.0664	117.0659	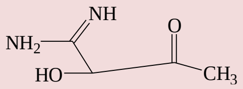
$C_4H_7NO_2$	TP-II-5	102.0555	102.0547	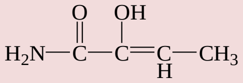
$C_{10}H_{11}N_3O_4S$	TP-III-1	284.0341	284.0340	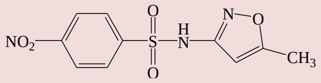
$C_9H_9N_3O_4S$	TP-IV-1	256.0392	256.0379	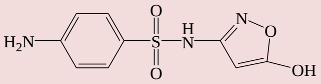

Supplementary Table 5 | The catalytic performance comparison of recently reported heterogeneous catalysts for TOC removal in SMX degradation.

Sample	Contaminant (10^{-6} M)	H_2O_2 (10^{-3} M)	Catalyst (mg L^{-1})	TOC removal (%)	Ref.
CX/CoFe	0.5 (mg L^{-1})	500 (mg L^{-1})	80	42.0	[2]
Sch@BC	10 (mg L^{-1})	2	1000	45.9	[3]
$Bi_2Fe_4O_9$	1.5	70	200	52.5	[4]
Magnetite	5 (mg L^{-1})	25 (mg L^{-1})	5	54.0	[5]
WO_3/Fe^{II}	30	0.06	1000	54.0	[6]
Fe@MesoC	20 (mg L^{-1})	3	200	54.5	[7]
Fe@N-C-800	50	2	50	75.4	this work

* 1 mg L^{-1} = 3.9×10^{-6} M for SMX, 1 mg L^{-1} = 29×10^{-6} M for H_2O_2.

Supplementary Table 6 | The catalytic performance comparison of recently reported heterogeneous catalysts for contaminant degradation in terms of K_{obs} value and H_2O_2 utilization efficiency.

Sample	Contaminant	Catalyst (mg L^{-1})	K_{obs} value (min^{-1})	H_2O_2 utilization efficiency (%)	Ref.
nZVCu-Cu^{II}-rGO	2-CP	400	0.038	63.0	[8]
Fe_3O_4/CeO_2	4-CP	2000	0.110	79.2	[9]
$BiFeO_3$	RhB	500	0.029	64.4	[10]
d-TiCuAl-SiO_2	BPA	800	0.062	80.0	[11]
γ -Cu-Al_2O_3	BPA	18	0.015	54.5	[12]
$CuFeO_2$	BPA	1000	0.047	57.8	[13]
$LaCu_{0.5}Fe_{0.5}O_{3-\delta}$	BPA	1000	0.021	70.4	[14]
CQDS-Fe^{III}	CIP	18	0.073	51.0	[15]
Fe@N-C-800	SMX	50	0.818	84.1	this work

* Thus far, there are few reports on the removal of SMX by heterogeneous catalyst/H_2O_2 system, we therefore selected other pollutants to compare the performance of the catalyst in terms of K_{obs} values and H_2O_2 utilization efficiencies.

Supplementary Table 7 | The catalytic performance comparison of recently reported N-doped carbon-supported metal composites in terms of K_{obs} value and TOC removal.

Sample	Contaminant	Oxidant	Catalyst (mg L^{-1})	K_{obs} (min^{-1})	TOC removal (%)	Ref.
FeCo-NC	BPA	PMS	100	1.252	/	[16]
Cu_1/NG	BPA	PDS	100	1.428	/	[17]
Co-N_2	BPA	PMS	200	0.695	72.9	[18]
SA-Cr/PN-g-C_3N_4	BPA	H_2O_2	200	0.096	/	[19]
Fe_1/CN	4-CP	PMS	500	0.53	59.2	[20]
Co-N-CNTs	SMX	PMS	100	0.157	/	[21]
Co-SA	CIP	PMS	200	0.105	60.0	[22]
FeCN	BPA	PAA	150	0.75	58.3	[23]
Fe-N_4-PC	SMX	PMS	30	0.21	72.8	[24]
Fe@N-C-800	SMX	H_2O_2	50	0.818	75.4	this work

Supplementary Table 8 | Operating details of HPLC

Item	Parameter
Flow rate (mL min^{-1})	2
Wavelength (nm)	190 ~ 800
Chromatographic column	HPLC ® BEH C18 1.7 µm, 2.1 × 100 mm Column
Temperature (°C)	25
Sample volume (µL)	20
Pressure (psi)	~ 2200

Supplementary Table 9 | Detection wavelength and mobile phase of different organic pollutants.

Category	Pollutant	Structural formula	Mobile phase	Wavelength
Pharmaceuticals	SMX	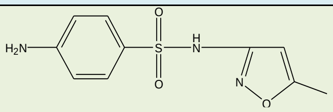	Acetonitrile: Formic acid (0.1%) = 60:40	265 nm
	CIP	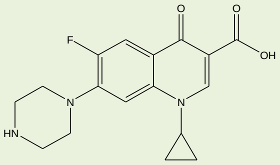	Acetonitrile: Water = 20:80	278 nm
	ENR	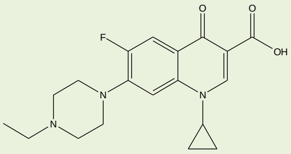	Acetonitrile: Water = 20:80	271 nm
	CBZ	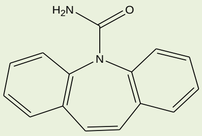	Acetonitrile: Water = 55:45	210 nm
	DCF	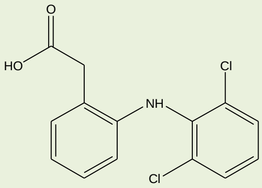	Methanol: Formic acid (0.1%) = 75:25	271 nm
	ATZ	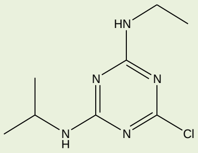	Acetonitrile: Water = 70:30	222 nm
Phenolic compounds	BPA	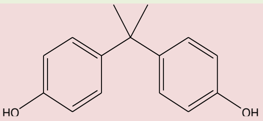	Methanol: Water = 60:40	276 nm
	Phenol	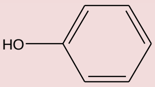	Acetonitrile: Water = 70:30	270 nm
	4-CP	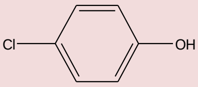	Methanol: Water = 30:70	280 nm
	PNP	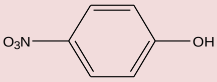	Methanol: Water = 30:70	254 nm
	TCP	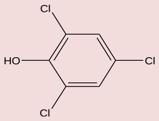	Methanol: Water = 30:70	290 nm

Supplementary Table 10 | Maximum absorption wavelength of various dyes.

Category	Dyes	Structure	Wavelength
Organic dyes	Acid orange 7 (AO7)	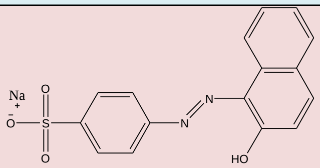	484 nm
	Reactive red M-3BE (RR M-3BE)	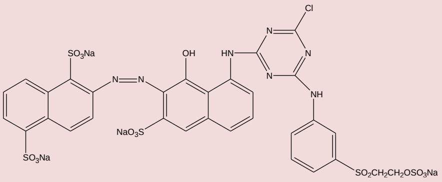	542 nm
	Rhodamine B (Rh B)	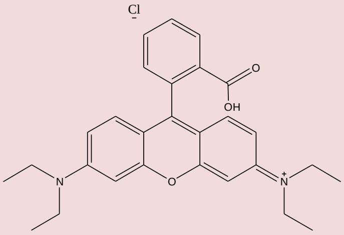	525 nm
	Methylene blue (MB)	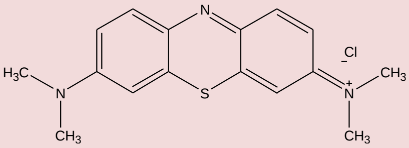	664 nm

References

1. Zhang, C. et al. $CuSO_4/H_2O_2$-induced rapid deposition of polydopamine coatings with high uniformity and enhanced stability. *Angew. Chem. Int. Ed*. **55**, 3054-3057 (2016).
2. Ribeiro, RS. et al. Magnetic carbon xerogels for the catalytic wet peroxide oxidation of sulfamethoxazole in environmentally relevant water matrices. *Appl. Catal. B-Environ.* **199**, 170−186 (2016).
3. Yang, Z. et al. Biosynthesized schwertmannite@biochar composite as a heterogeneous Fenton-like catalyst for the degradation of sulfanilamide antibiotics. *Chemosphere* **266**, 129175 (2021).
4. Hu, ZT. et al. Enhanced $BiFeO_3/Bi_2Fe_4O_9/H_2O_2$ heterogeneous system for sulfamethoxazole decontamination: System optimization and degradation pathways. *J. Colloid Interf. Sci.* **577**, 54−65 (2020).
5. Munoz, M. et al. Antibiotics abatement in synthetic and real aqueous matrices by H_2O_2/natural magnetite. *Catal. Today* **313**, 142−147 (2018).
6. Tian, Y. et al. Photo-Fenton-like degradation of antibiotics by inverse opal WO_3 co-catalytic Fe^{2+}/PMS, Fe^{2+}/H_2O_2 and Fe^{2+}/PDS processes: A comparative study. *Chemosphere* **288**, 132627 (2022).
7. Tang, J., Wang, J. Fenton-like degradation of sulfamethoxazole using Fe-based magnetic nanoparticles embedded into mesoporous carbon hybrid as an efficient catalyst. *Chem. Eng. J.* **351**, 1085−1094 (2018).
8. Lyu, L. et al. Enhanced polarization of electron-poor/rich micro-centers over nZVCu-Cu (II)-rGO for pollutant removal with H_2O_2. *J. Hazard. Mater.* **383**, 121182 (2020).
9. Xu, L., Wang, J. Magnetic nanoscaled Fe_3O_4/CeO_2 composite as an efficient Fenton-like heterogeneous catalyst for degradation of 4-chlorophenol. *Environ. Sci. Technol.* **46**, 10145−10153 (2012).
10. Luo, W. et al. Efficient removal of organic pollutants with magnetic nanoscaled $BiFeO_3$ as a reusable heterogeneous Fenton-like catalyst. *Environ. Sci. Technol.* **44**, 1786−1791 (2010).
11. Lyu, L. et al. Galvanic-like cells produced by negative charge nonuniformity of lattice oxygen on d-TiCuAl–SiO_2 nanospheres for enhancement of Fenton-catalytic efficiency. *Environ. Sci. Nano* **3**, 1483−1492 (2016).
12. Xie, Z. et al. Novel Fenton-like catalyst γ-Cu-Al_2O_3-$Bi_{12}O_{15}Cl_6$ with electron-poor Cu centre and electron-rich Bi centre for enhancement of phenolic compounds degradation and H_2O_2 utilization: The synergistic effects of σ-Cu-ligand, dual-reaction centres and oxygen vacancies. *Appl. Catal. B-Environ.* **253**, 28−40 (2019).
13. Zhang, X. et al. Degradation of bisphenol A by hydrogen peroxide activated with $CuFeO_2$ microparticles as a heterogeneous Fenton-like catalyst: efficiency, stability and mechanism. *Chem. Eng. J.* **236**, 251−262 (2014).
14. Pan, K. et al. Oxygen vacancy mediated surface charge redistribution of Cu-substituted $LaFeO_3$ for degradation of bisphenol A by efficient decomposition of H_2O_2. *J. Hazard. Mater.* **389**, 122072 (2020).
15. Zhang, T. et al. Overcoming acidic H_2O_2/Fe(II/III) redox-induced low H_2O_2 utilization efficiency by carbon quantum dots Fenton-like catalysis. *Environ. Sci. Technol.* **56**, 2617−2625 (2022).

16. Li, X. et al. Single cobalt atoms anchored on porous N-doped graphene with dual reaction sites for efficient Fenton-like catalysis. *J. Am. Chem. Soc.* **140**, 12469−12475 (2018).
17. Wang, B. et al. A site distance effect induced by reactant molecule matchup in single-atom catalysts for Fenton-like reactions. *Angew. Chem.* **134**, e202207268 (2022).
18. Liang, X. et al. Coordination number dependent catalytic activity of single-atom cobalt catalysts for Fenton-like reaction. *Adv. Funct. Mater.* **3**, 2203001 (2022).
19. Chen, F. et al. Molecular engineering toward pyrrolic N-rich M-N_4 (M = Cr, Mn, Fe, Co, Cu) single-atom sites for enhanced heterogeneous Fenton-like reaction. *Adv. Funct. Mater.* **31**, 2007877 (2021).
20. Zhang, LS. et al. Carbon nitride supported high-loading Fe single-atom catalyst for activation of peroxymonosulfate to generate $^1O_2$ with 100% selectivity. *Angew. Chem. Int. Ed.* **60**, 21751–21755 (2021).
21. Miao, J. et al. Spin-state-dependent peroxymonosulfate activation of single-atom M–N moieties via a radical-free pathway. *ACS Catal.* **11**, 9569–9577 (2021).
22. Mi, X. et al. Almost 100% peroxymonosulfate conversion to singlet oxygen on single-atom CoN_{2+2} sites. *Angew. Chem.* **133**, 4638–4643 (2021).
23. Chen, F. et al. Single-atom iron anchored tubular g-C_3N_4 catalysts for ultrafast Fenton-like reaction: Roles of high-valency iron-oxo species and organic radicals. *Adv. Mater.* **34**, 2202891 (2022).
24. Wang, J. et al. Facile synthesis of atomic Fe-N-C materials and dual roles investigation of Fe-N_4 sites in Fenton-like reactions. *Adv. Sci.* **8**, 2101824 (2021).
